# Adaptive cut reveals multiscale complexity in networks

**DOI:** 10.1093/pnasnexus/pgag344

**Published:** 2026-10-06

**Authors:** Louis Boucherie, Yong-Yeol Ahn, Sune Lehmann

**Affiliations:** Department of Applied Mathematics and Computer Science, Technical University of Denmark, Lyngby, Denmark; Copenhagen Center for Social Data Science (SODAS), University of Copenhagen, Copenhagen, Denmark; School of Data Science, University of Virginia, Charlottesville, VA, USA; Department of Applied Mathematics and Computer Science, Technical University of Denmark, Lyngby, Denmark; Copenhagen Center for Social Data Science (SODAS), University of Copenhagen, Copenhagen, Denmark

**Keywords:** hierarchical clustering, community detection, dendrograms, networks, MCMC

## Abstract

Hierarchical clustering and community detection are important problems in machine learning and complex network analysis. A common approach to identify clusters is to simply cut dendrograms at some threshold. However, single-level cuts are often suboptimal in terms of capturing underlying structure in the data, especially when the dendrogram is unbalanced. In this article, we present the adaptive cut, a method that uses the hierarchical structure of dendrograms through multilevel cuts to overcome the limitations of single-level approaches. The adaptive cut optimizes an objective function using a Markov chain Monte Carlo with simulated annealing, resulting in better partitions. We demonstrate the adaptive cut through applications to link clustering and modularity optimization, but note that the method is applicable to any clustering task that relies on a dendrogram and an objective function. Beyond the adaptive cut, we introduce the balancedness score, an information-theoretic metric that quantifies how balanced a dendrogram is. Balancedness predicts the potential benefits of using multilevel cuts. For the community detection examples, we evaluate our method on more than 200 real-world networks and multiple synthetic datasets, demonstrating improvements in partition density and modularity over traditional single-cut approaches. We also show the generality of the adaptive cut by applying it across hierarchical clustering techniques and objective functions. The adaptive cut improves clustering outcomes across a range of hierarchical clustering tasks.

Significance Statement Hierarchical clustering produces tree-structured data representations across nearly every discipline, from gene expression and protein interactions to social networks and food webs. Extracting groups from these trees relies on cutting the dendrogram at a single level, which fails when the data has structure at multiple scales, as in many biological and social systems. We introduce the adaptive cut, which replaces this single-level heuristic with a multilevel optimization, and a companion balancedness metric that diagnoses when multilevel cuts are most needed. Across over 200 real-world networks, adaptive cut recovers higher-quality partitions, with the largest gains where hierarchical heterogeneity makes single-level cuts most inadequate. Because dendrograms are a ubiquitous representation, the method is a general-purpose postprocessing step for any hierarchical clustering pipeline.

## Introduction

Identifying groups of objects that are closely related, which is commonly referred to as “clustering” or “community detection” in the case of networks, is an important problem in many scientific fields. A plethora of clustering methods have been proposed in the literature (1–5). Community detection and cluster analysis methods frequently produce hierarchies, reflecting the multiscale nature found in many datasets (6–8). The end product of hierarchical clustering is a merge tree (or dendrogram if binary) originating from leaves containing data elements and culminating at the root, which encompasses the entire dataset. Dendrograms are a common feature of both clustering and community detection methods. In this article, we focus on hierarchical clustering and community detection in complex networks, in particular the Link Clustering algorithm (9) and the Louvain optimization of modularity (6). However, our approach can be applied to any clustering process that produces a dendrogram and an independent function of clustering quality.

The most common method for obtaining clusters (or communities) is to cut the dendrogram at a single level or a constant height cutoff value (10–12). There are several methods for selecting this value, including optimizing an objective function (9, 13), obtaining a specific number of clusters (through the elbow method or the silhouette method) (12), or optimizing for clusters with high intracluster similarity and high intercluster differences (14). However, single-level cuts may not always identify the most useful clusters. In particular, many dendrograms are unbalanced (e.g. due to different levels of data density across a dataset), and a single-level cut cannot resolve the trade-off between over-aggregation in one part of the dendrogram and providing an excessively granular view in another part. Consequently, single-level cuts are incapable of optimally separating distinct clusters.

In this sense, single-level cuts are unable to use all of the information contained within the hierarchical structure of the dendrogram. In unbalanced dendrograms, choosing a single cut point can result in the formation of clusters with very different sizes. This can have a range of undesired downstream consequences. For example, statistical tests that assume equal cluster sizes may be unsuitable when employed in the analysis of unbalanced clusters (15, 16). Similarly, machine learning algorithms that rely on balanced training sets may underperform (17, 18).

Here, we propose the *adaptive cut*, a method for cutting dendrograms using a multilevel cut. Our approach improves a range of tasks that involve hierarchical tree structures. To demonstrate the robustness of our results, we evaluate the method on over 200 real networks. The adaptive cut optimizes an objective function along the dendrogram using a Markov chain Monte Carlo with a simulated annealing scheme. We demonstrate the generality of the method across two distinct use cases of community detection for network nodes and edges. The previous key approach to examine this has been based on a visualization tool for exploring clustering scenarios at different cut levels (19). However, this approach depends on expert knowledge and does not provide a quantitative measure. Another approach (20) proposes a heuristic based on dendrogram shape, akin to the silhouette coefficient (21).

We also present a new measure of dendrogram balance. Numerous tree balance indices have been proposed in the literature (22–25). Classical indices such as the Colless and Sackin indices measure the topological shape of the tree, while more recent information-theoretic indices (24) quantify the entropy of subtree sizes. The entropy-based normalization closest to ours is the normalized tree balance of (22), which averages a local Shannon entropy over the internal nodes of the dendrogram. Our index, named *balancedness*, differs from these approaches in that it is defined at each *similarity level* of the dendrogram rather than at each branching event. This level-wise formulation captures how balanced the partition is at each hierarchical scale, making it directly relevant as a predictor of adaptive cut improvement (a head-to-head comparison with the index of (22) on the 202-network catalog is reported in Section S6.4). Our index is computationally efficient and satisfies the axioms required for membership in the class of robust, universal tree balance indices (24) (see Balancedness axioms section, for the proof).

Existing work on community detection also connects to our method. Markov chain Monte Carlo with simulated annealing has been employed for community detection in networks to optimize modularity (26, 27), description length (8), or fit stochastic block models (SBMs) (28). These approaches optimize community splits directly and as such do not use the tree structure to optimize the objective function. They operate on a large state space, making convergence to the optimal partition challenging and computationally expensive. By using an underlying tree structure, adaptive cut reduces the size of the state space and enables a more efficient optimization of the objective function and faster convergence to an optimal solution.

## Balancedness

### Definition

We begin by introducing the *balancedness* score, an information theory based metric that quantifies the balance of a dendrogram. The balancedness metric compares the actual branching structure of a dendrogram (real entropy) with both a perfectly balanced scenario (maximal entropy) and a highly skewed one (minimal entropy) to quantify how balanced a particular dendrogram is. At each level *l* of the dendrogram, the partition of the *n* leaves in each of the *k* branches is noted as follows:


(1) $$\pi_{l}=\{B_{1},B_{2},\ldots ,B_{k}\},$$

where $B_{i}$ represents the set of leaves having branch $B_{i}$ as an ancestor. To normalize the entropy at each level, we compute the upper and lower bounds on Shannon entropy for any partition of *n* elements into *k* nonempty groups. The *maximal entropy* is attained by the most balanced partition, where nmodk groups have size ⌈n/k⌉ and the remaining groups have size ⌊n/k⌋:


(2) $$H_{\max}(k,n)=-\sum_{i=1}^{k}\frac{n_{i}}{n}\log_{2}\;\left(\frac{n_{i}}{n}\right),$$

where $n_{i}=\lceil n/k\rceil$ for i≤nmodk and $n_{i}=\lfloor n/k\rfloor$ otherwise. Note that $H_{\max}(k,n)=\log_{2}\;k$ only when *k* divides *n* evenly. The *minimal entropy* is attained by the most skewed partition, where one group contains n−(k−1) elements and the remaining k−1 groups each contain one element:


(3) $$\begin{array}{rl} H_{\min}(k,n) & =-\sum_{i=1}^{k-1}\frac{1}{n}\log_{2}\;\left(\frac{1}{n}\right)\\ & \;-\frac{n-(k-1)}{n}\log_{2}\;\left(\frac{n-(k-1)}{n}\right). \end{array}$$

We also determine the realized entropy of the leaf partition $\pi_{l}$ at level *l* as follows:


(4) $$H_{\;}(\pi_{l})=-\sum_{i=1}^{\left|\pi_{l}\right|}p(B_{i})\log_{2}\;p(B_{i}),$$

where $p(B_{i})=|B_{i}|/n$. The balancedness score is the average, over all levels, of the ratio between the realized entropy minus the minimal entropy and the maximum entropy minus the minimal entropy across all levels (we write $H_{\max}(\pi_{l})$ and $H_{\min}(\pi_{l})$ for $H_{\max}(k,n)$ and $H_{\min}(k,n)$ evaluated at the level-*l* partition, with $k=|\pi_{l}|$ branches),


(5) $$B=\frac{1}{L}\sum_{i=1}^{L}\frac{H_{\;}(\pi_{l})-H_{\min}(\pi_{l})}{H_{\max}(\pi_{l})-H_{\min}(\pi_{l})}.$$

The balancedness metric ranges from 0 (unbalanced dendrogram, Fig. 1A) to 1 (perfectly balanced dendrogram, Fig. 1C). The balancedness score also satisfies the axioms of a robust and universal measure of tree balance (24). Balancedness can be computed in O(n) with a single pass over levels.

**Figure 1:**
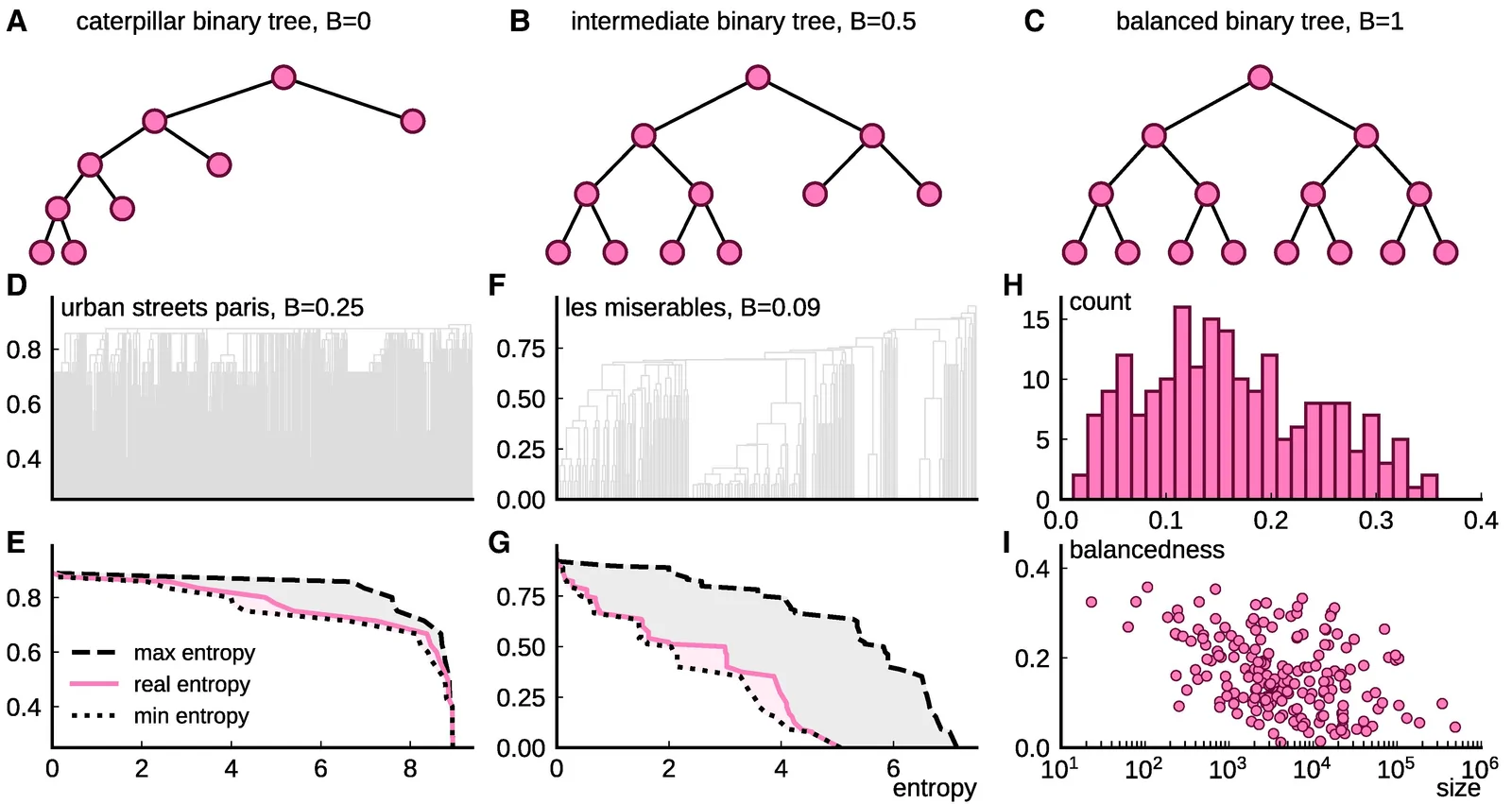
Explanation of the balancedness measure. A–C) Illustrations of different tree structures: A) an unbalanced caterpillar tree, B) an intermediate tree, and C) a balanced tree. D) Dendrogram of the urban street network of Paris based on link similarities (9). This network sits in the top quartile of our 202-network catalog (B=0.25, see h)); see e) for the per-level entropy curves. e) The Shannon entropy $H(\pi_{l})$ of the partition at each dendrogram level, alongside its theoretical upper bound $H_{\max}$ and lower bound $H_{\min}$. The *y*-axis is shared with the dendrogram in (d). The balancedness score (Eq. 5) corresponds to the average normalized position of the entropy curve between the two bounds. f) Dendrogram of the “Les Miserables” character network based on link similarities (9). The dendrogram is highly unbalanced, as shown in (g). g) The entropy curves for the Les Miserables network, computed as in (e). h) The distribution of balancedness scores for 202 real networks. i) The balancedness metric plotted against network size (number of edges *M*). There is a negative correlation between *B* and size (Pearson r=−0.21), indicating that larger networks tend to produce more unbalanced dendrograms under link clustering.

### Examples

To illustrate our metric, we examine the balancedness score of two real networks. Figure 1D,F shows the dendrograms obtained by link clustering of the street network of Paris and the character network of Les Miserables, respectively. The Paris network sits in the top quartile of our catalog (B=0.25, ranked 36 of 202, see Fig. 1H), whereas the Les Miserables network is highly unbalanced. To determine the balancedness of each dendrogram, we compute the maximum, minimum, and realized entropy values at each level as defined in Eqs. 2–5. These values are displayed alongside each dendrogram in Fig. 1E,G. The balancedness score corresponds to the average normalized position of the realized entropy curve between the upper and lower bounds.

A majority of real-world networks yield unbalanced dendrograms, as illustrated in Fig. 1H. Figure 1I shows a negative correlation between balancedness and network size (Pearson r=−0.21), indicating that larger networks tend to produce more unbalanced dendrograms under link clustering. This further motivates the adaptive cut, as larger networks are those that benefit most from multilevel cuts.

## Adaptive cut

To identify the multilevels of the adaptive cut, we optimize an objective function over the partitions (or cluster membership). We employ a Markov chain Monte Carlo (MCMC) approach to optimize the objective function *f* within a finite search space *X*, using the softmax distribution


(6) $$\pi^{⋆}(x)=\frac{e^{\;f(x)/T}}{Z},$$

where *x* represents a state within the search space *X*, *T* denotes the temperature parameter, and *Z* is the partition function.

Because moves between adaptive cuts change the number of clusters, the sampler is a reversible-jump MCMC (RJMCMC) (29) with an asymmetric proposal. The Markov chain walks up or down the dendrogram: an up-move merges a selected cluster with its dendrogram sibling (collapsing the sibling’s descendants when needed), and a down-move splits a selected cluster by sampling an adaptive cut of its subtree. Each move is accepted or rejected with a Metropolis–Hastings probability that includes the proposal-density ratio; this guarantees detailed balance with respect to $\pi^{⋆}$ even though up- and down-moves are not symmetric. The chain is ergodic because any partition can be reached from any other via a sequence of up- and down-moves. The full proposal mechanism, transition probabilities $Q_{x\to y}$, Metropolis–Hastings acceptance ratio, and simulated-annealing cooling schedule are given in Methods; the formal proofs of detailed balance and ergodicity are in Section S2. We initialize the chain at the single-level cut returned by the underlying clustering algorithm: starting at a known local maximum dramatically shortens mixing time relative to a random partition (30).

## Results

### Link Clustering with toy network models

The Link Clustering method (9) uses hierarchical clustering based on link similarity (Jaccard index of neighbors) to create a dendrogram, where each link is a leaf, and branches represent link communities. Communities are extracted by cutting the dendrogram at a specific similarity threshold, allowing nodes to belong to multiple overlapping communities (see Link similarity and dendrogram construction section). To identify the most relevant communities, the method introduces partition density *D*, which measures link density within communities and does not suffer from resolution limits (9, 31, 32). The optimal cut is determined by maximizing *D* along the levels of the dendrogram (see Partition density section).

However, determining the best cut level can be challenging. The adaptive cut method optimizes *D* further, potentially revealing more accurate community structures, especially in networks with communities of varying sizes and densities. This approach addresses the limitations of a single-level cut by better capturing complex network structures (Fig. 2C).

**Figure 2:**
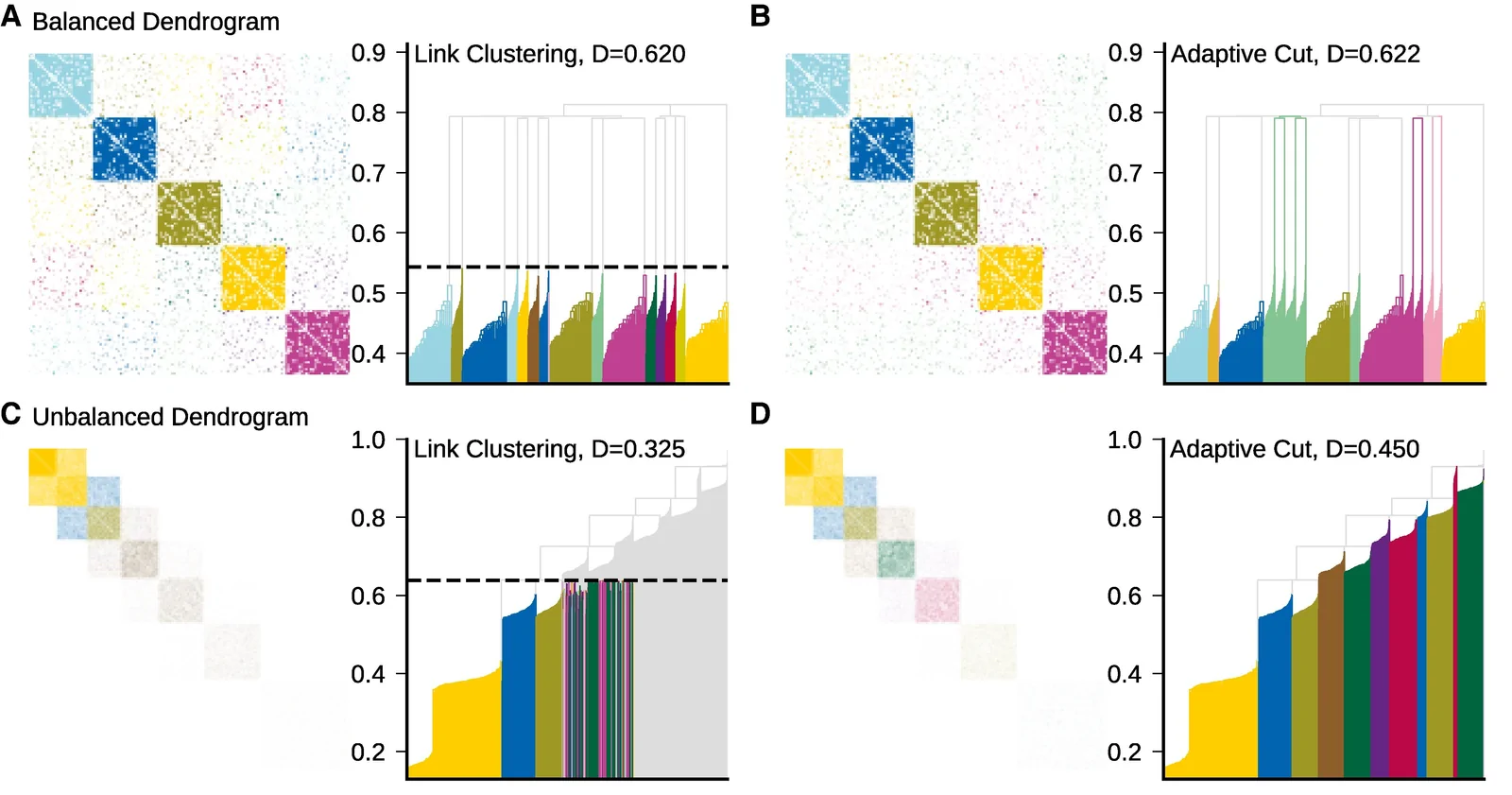
Comparison between link clustering and adaptive cut. A) The adjacency matrix of a SBM network, with nodes colored according to edge communities identified by the link clustering method (9). B) The corresponding dendrogram for the same network, with partitions or communities defined by the single-level link clustering cut (9). The similarity level at which the cut is made is indicated by the dashed line. C,D) Similar network to (A) and (B), but using an adaptive cut method instead of link clustering.

#### Varying density stochastic block model

To illustrate the limitations of the single-level cut, we focus on two toy networks with a given community structure, a SBM (33, 34) and a varying density stochastic block model (VDSBM) that we introduce. We simplify the SBM by assuming that the probability of an edge between two nodes depends only on whether the nodes belong to the same community or different communities (see the adjacency matrix Fig. 2A). The model is described by the following equation:


(7) $$P(A_{ij}=1|z_{i}=z_{j})=\theta_{\mathrm{intra}},\;P(A_{ij}=1|z_{i}\neq z_{j})=\theta_{\mathrm{inter}},$$

where $A_{ij}$ is the adjacency matrix entry for nodes *i* and *j*, where $A_{ij}=1$ indicates the presence of an edge between these nodes, and $A_{ij}=0$ otherwise. The variables $z_{i}$ and $z_{j}$ denote the community assignments of nodes *i* and *j*, respectively. The term $\theta_{\mathrm{intra}}$ represents the probability of an edge between any two nodes in the same community, while $\theta_{\mathrm{inter}}$ represents the probability of an edge between nodes in different communities. In this model, $\theta_{\mathrm{intra}}$ is the same for all communities, and $\theta_{\mathrm{inter}}$ is the same for all pairs of different communities. Our VDSBM is the same, except that the $\theta_{\mathrm{intra}}$ decreases and the $\theta_{\mathrm{inter}}$ also decreases. In the VDSBM, we generalize the traditional SBM by varying the intracommunity and intercommunity densities. This approach allows the creation of communities with different internal structures and varying degrees of connectivity between them. In particular, the intra- and intercommunity densities decrease across communities (see Varying density stochastic block model section).

The SBM (adjacency matrix in Fig. 2A) exhibits clear communities of identical size and density, exhibiting identical similarities with respect to link clustering. The single-level cut is capable of distinguishing between the communities (see Fig. 2A), while the adaptive cut does not offer a superior partitioning (see Fig. 2B). The partition densities of the two cuts are close, D=0.620 for link clustering and D=0.622 for the adaptive cut. Conversely, although the VDSBM still exhibits a clear community structure, the single-level cut is unable to provide a partition that reflects these communities. This is due to the inability of a single-cut to capture communities with varying densities. The adaptive cut improves performance, with a value of D=0.45, in comparison to the single-level cut, which has a value of D=0.325 (Fig. 2C,D). The communities are aligned with the blocks of the adjacency matrix, as illustrated in Fig. 2D. Finally, it can be observed that the SBM dendrogram is more balanced than the dendrogram of the VDSBM. This is evidenced by the respective balancedness values of B=0.23 and B=0.03.

#### Community structure evaluation

In Fig. 3A, we compare the log-scale average cluster size produced by each method with the true cluster size. The Link Clustering method has a smaller average community size because it leaves many links isolated in singleton communities (21,254). This observation is due to the tendency for the single-level cuts of unbalanced networks to “over-partition.” In contrast, the adaptive cut method successfully merges these small communities into larger communities—shifting its mean cluster size much closer to the ground truth, reflecting a more accurate representation of the structure of the network, as can be seen in the adjacency matrix (see Fig. 2D).

**Figure 3:**
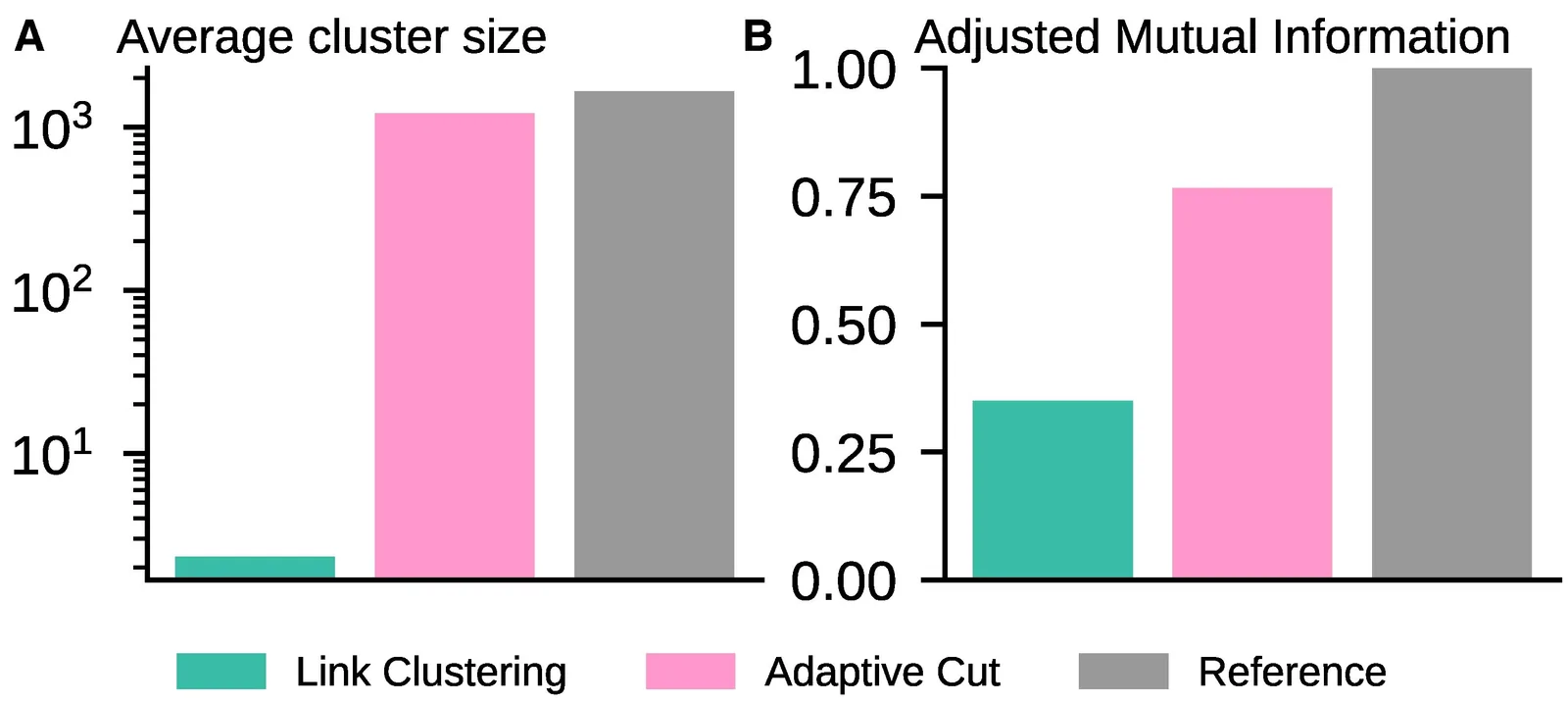
VDSBM network: comparison of average cluster size and recovery accuracy. A) Comparison of the average cluster size (log-scale) for Link Clustering, the adaptive cut, and the ground truth. Link Clustering has many singleton communities that yield a smaller average size, whereas the adaptive cut merges the singletons into clusters, so the average cluster size matches the ground truth better. B) Adjusted mutual information between the detected partitions and the ground truth assignments. The adaptive cut has higher mutual information with ground truth than Link Clustering, indicating a better community detection.

Figure 3B shows the mutual information between each detected partition and the true cluster assignments. The adaptive cut has a higher mutual information with the ground truth than Link Clustering, demonstrating that its multilevel cutting scheme recovers the underlying community structure more accurately than a single-level cut.

### Link Clustering with real networks examples

We compare the Link Clustering and adaptive cut across 202 real-world networks to evaluate (a) the typical improvement and (b) the influence of balancedness on the outcome of the community detection. Figure 4 presents the extent to which adaptive cut enhances partition density (expressed as a percentage) relative to the balancedness of each dendrogram. Our findings indicate that as the balancedness of a network decreases, the likelihood of achieving significant improvements with adaptive cut increases. Across the 202 networks the adaptive cut improves *D* over the single-level cut on 195 networks (mean +34%, median +21%), with the largest gains concentrated at low *B*: networks with B<0.15 have a ≥75% chance of ≥10% improvement under adaptive cut (Section S6). A deterministic greedy hill-climber on the same dendrogram and the same objective recovers about 21 of those 34 percentage points; the remaining ∼13 points come from the RJMCMC’s ability to escape greedy local optima via the geometric-penalty multilevel proposal (Section S3). This relationship holds across network domains, including economic, transportation, informational, biological, and social networks (the full per-network results, with domain, size, $D_{\mathrm{lc}}$, $D_{\mathrm{ac}}$, and source, are listed in Table S11 (Section S7)). The robustness of this finding to the choice of linkage method (single, average, weighted, Ward) is examined in Section S4, where we show that the adaptive cut improves partition density on the large majority of the eighty network–linkage combinations from a sample of 20 catalog networks (the only exceptions are two near-degenerate runs), confirming that the method is agnostic to the dendrogram construction method.

**Figure 4:**
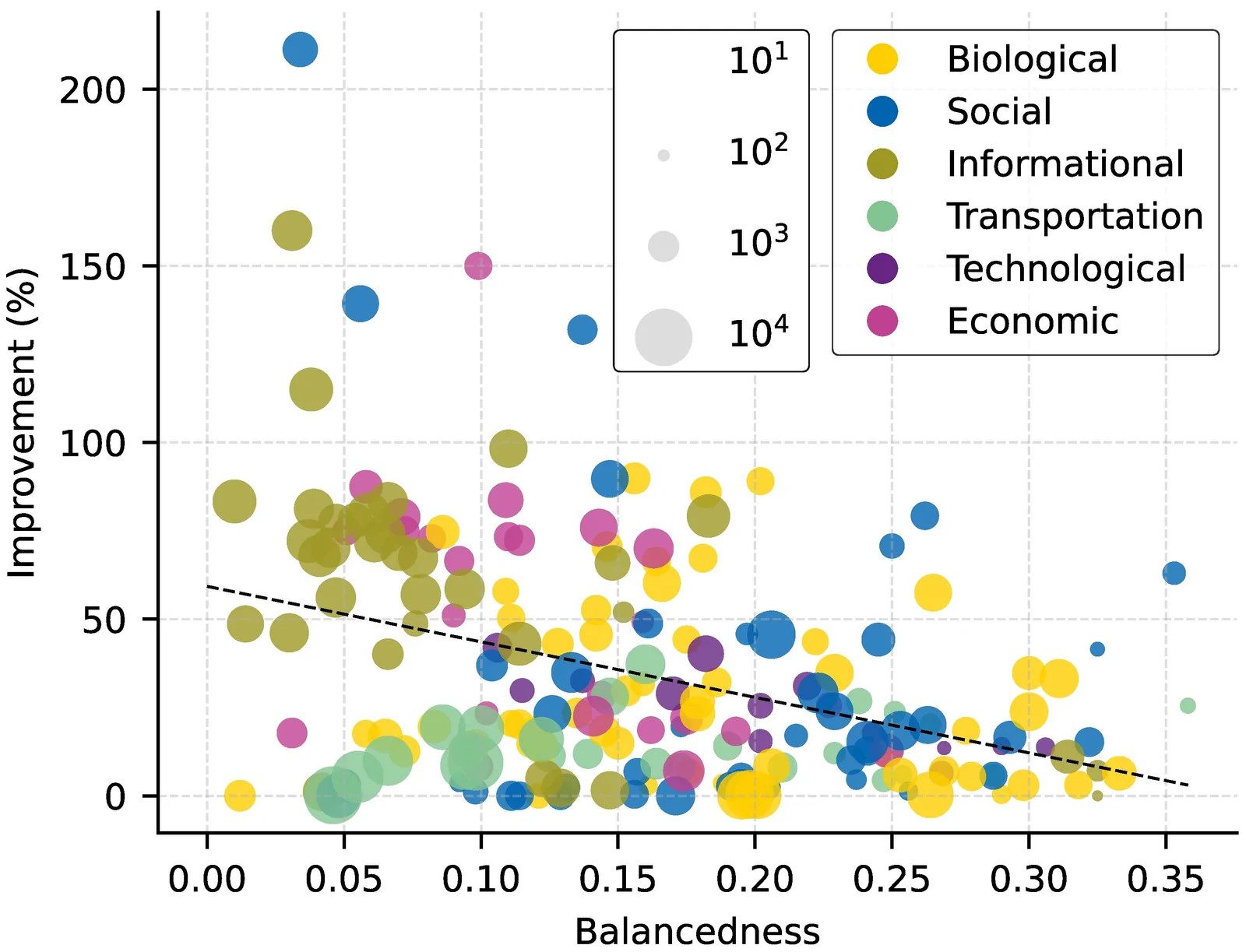
Improvement (in %) of the partition density between the single-level cut (Link Clustering (9)) and the multilevel adaptive cut as a function of the dendrogram balancedness. The color of the symbols indicates the domain of the network, and the size indicates the number of nodes in the network.

### Louvain with real network examples

The Louvain method (6) is a popular algorithm for community detection that optimizes modularity to identify community structures in networks. It operates iteratively, initially assigning each node to its own community and then repeatedly merging communities to maximize modularity gains. However, this greedy optimization can lead to the algorithm getting trapped in local maxima, potentially missing the globally optimal community structure. The standard Louvain method also does not produce a complete dendrogram that represents the full hierarchy of community merges, as it stops merging when no further modularity gain is possible.

To integrate the adaptive cut into the Louvain method, we modify the algorithm to construct a full dendrogram, extending beyond the point where modularity gain ceases. This is achieved by allowing merges that result in a modularity decrease, thereby capturing the entire hierarchical structure of the network from individual nodes up to the entire network as a single community. The MCMC then optimizes modularity over the resulting dendrogram using the sampler defined in Methods (Reversible-jump MCMC sampler section).

Our experiments on real-world networks demonstrate improvements in modularity when applying the adaptive cut to the Louvain method. For instance, in networks with unbalanced dendrograms, the adaptive cut effectively adjusts for imbalance by selecting cuts at different levels of the dendrogram, leading to partitions with higher modularity. Figure 5 shows the percentage improvement in modularity achieved by the adaptive cut compared to the original Louvain partition, plotted against the balancedness score of each network’s dendrogram. The results indicate that networks with lower balancedness scores benefit more from the adaptive cut, validating our approach.

**Figure 5:**
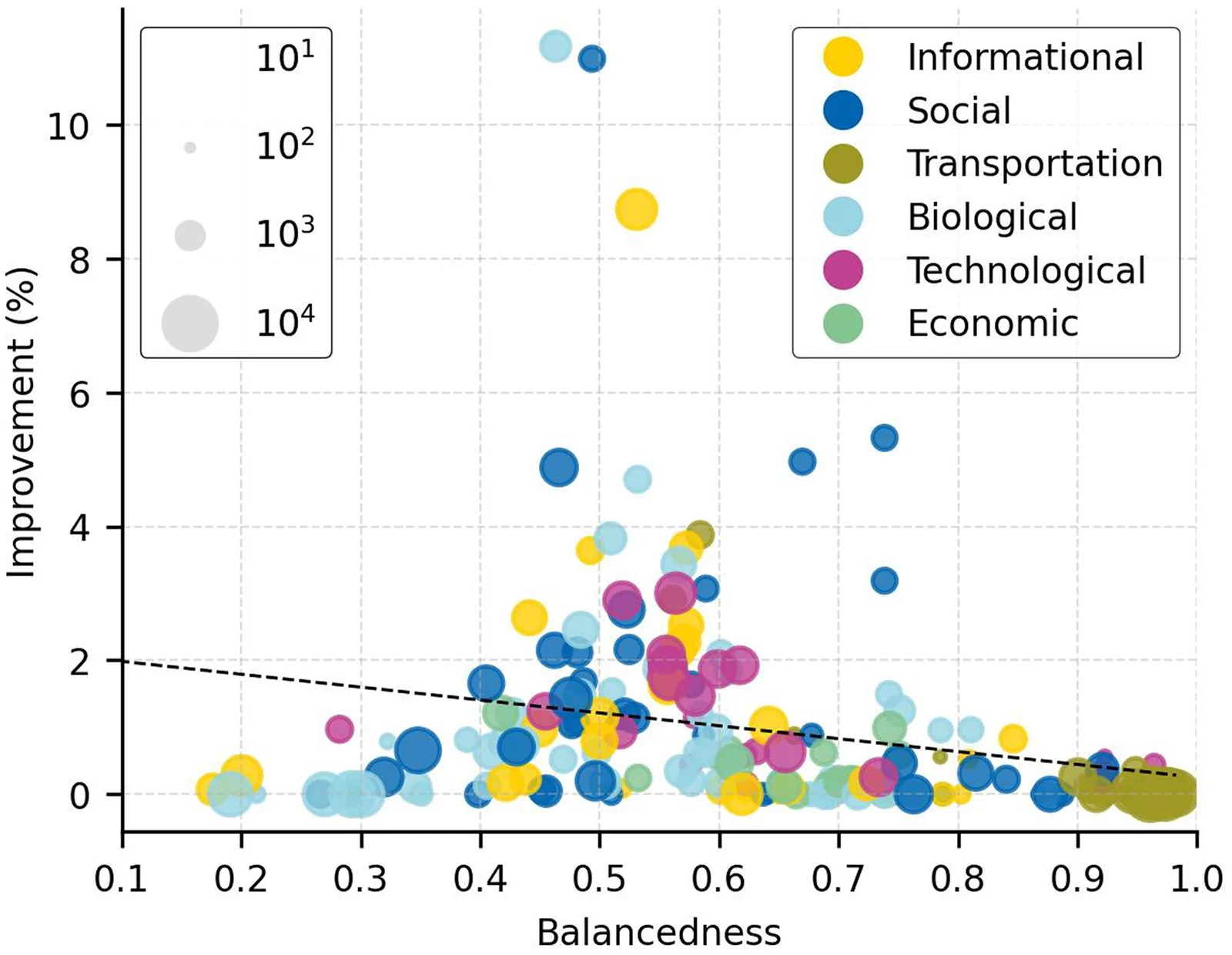
Improvement (in %) of the modularity between the single-level cut (Louvain) and the multilevel cut (Louvain + adaptive cut) as a function of balancedness. The symbol colors indicate the domain of the network, and the size the number of nodes in the network.

### Generalizing to any hierarchical clustering

The adaptive cut framework extends beyond network community detection and is applicable to any clustering task that produces a dendrogram. This generalization requires two main components: a method for constructing the dendrogram and an objective function to optimize.

For dendrogram construction, any hierarchical clustering algorithm can be used, such as single linkage (2), average linkage (35), or Ward’s method (36). These methods build a hierarchical tree by iteratively merging clusters based on a chosen linkage criterion.

An appropriate objective function is necessary to evaluate the quality of partitions at different levels of the dendrogram. Common choices include the Davies–Bouldin index (14), silhouette score (21), within-cluster sum of squares as in K-means clustering (37), or the ratio of intra-cluster to intercluster distances.

Once the dendrogram and objective function are defined, the adaptive cut is applied by optimizing the objective function using the reversible-jump Markov chain Monte Carlo sampler described in Methods (Reversible-jump MCMC sampler section). The chain explores partitions by moving up and down the dendrogram to find the partition that optimizes the objective function, with detailed balance and ergodicity guaranteed by the matched-support construction proved in Section S2.

To illustrate the generality of our method, we apply the adaptive cut to the classic two-circle toy dataset (38), which presents a challenging clustering problem due to its nonconvex cluster shapes. We construct a dendrogram using hierarchical clustering and use the within-cluster sum of squares as the objective function. Figure 6 compares the clustering results of the traditional single-level cut and the adaptive cut. While the single-level cut fails to separate the concentric circles, the adaptive cut successfully identifies the inherent cluster structure through multilevel cuts.

**Figure 6:**
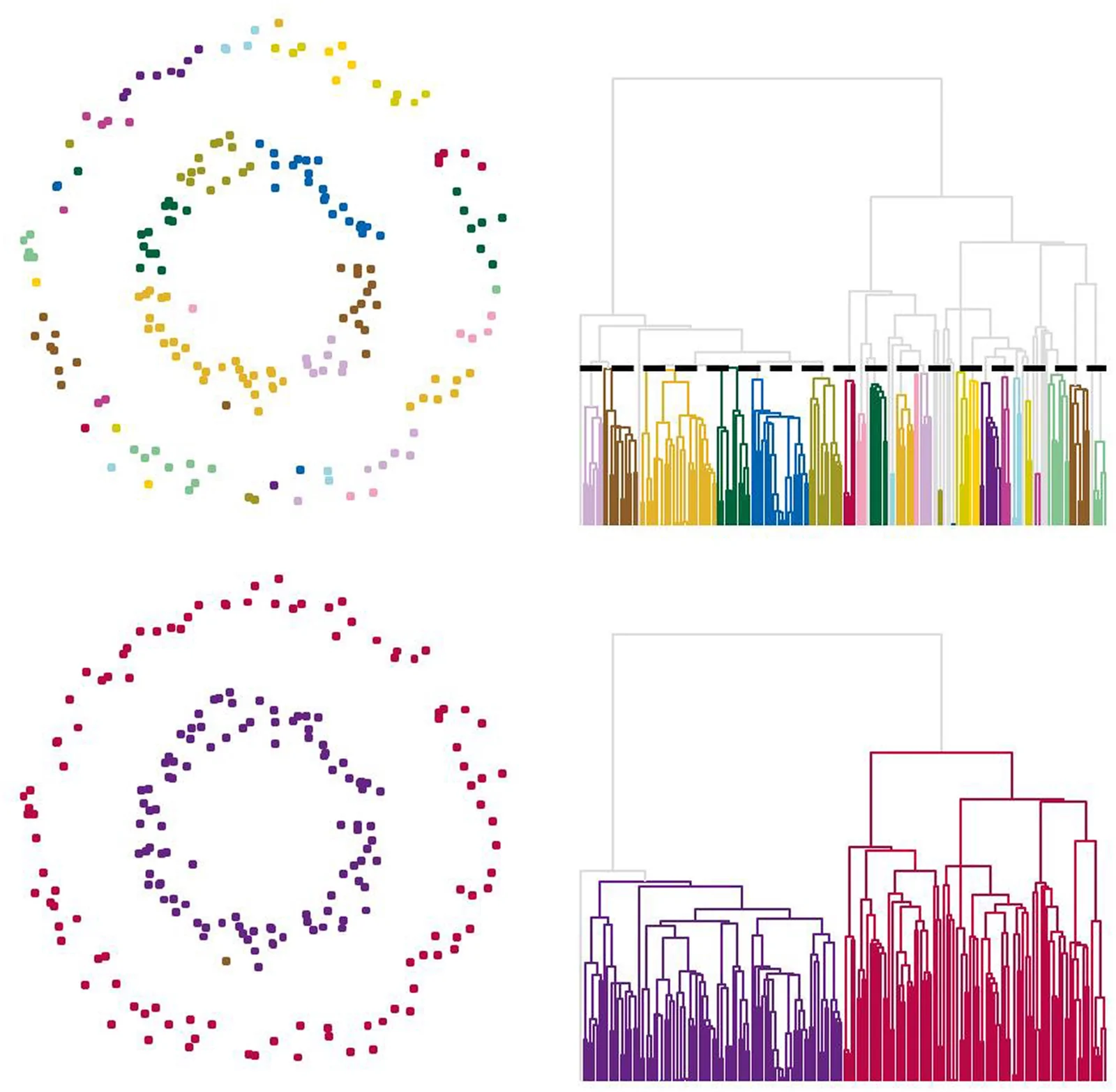
Comparison of clustering results on the two-circle dataset using a single-level cut and the adaptive cut. The adaptive cut identifies the two concentric clusters, which the single-level cut fails to separate.

## Methods

### Link similarity and dendrogram construction

To construct a dendrogram representing link communities (9), we first define the similarity between pairs of links. For an undirected, unweighted network, let n(i) denote the set of neighbors of node *i*. The similarity between two links $e_{ik}$ and $e_{\;jk}$ that share a common node *k* is the Jaccard index of the endpoint neighborhoods,


(8) $$S(e_{ik},e_{\;jk})=\frac{\left|n(i)\cap n(j)\right|}{\left|n(i)\cup n(j)\right|}.$$

For weighted and directed networks, this generalizes to the Tanimoto coefficient (39). Single-linkage hierarchical clustering on the resulting similarity matrix yields the dendrogram: each link starts as its own cluster and clusters are merged iteratively in decreasing order of similarity until a single cluster remains.

### Partition density

To identify a meaningful level of clustering within a dendrogram, we use the partition density *D* (9). Given a network with *M* links partitioned into *C* subsets, where subset *c* contains $m_{c}$ links spanning $n_{c}$ nodes, the link density of community *c* is


(9) $$D_{c}=\frac{m_{c}-(n_{c}-1)}{n_{c}(n_{c}-1)/2-(n_{c}-1)}.$$

The overall partition density is the link-weighted average,


(10) $$D=\frac{2}{M}\sum_{c}m_{c}\frac{m_{c}-(n_{c}-1)}{\left(n_{c}-2)(n_{c}-1\right)}.$$

*D* is free from the resolution limit (31, 32) and serves as the objective function maximized by the adaptive cut.

### Varying density stochastic block model

The VDSBM generalizes the standard SBM by allowing the intracommunity edge probability to vary across communities. For community *c* of size $k_{c}$ with per-node edge probabilities $\{p_{i}{\}}_{i\in c}$, the intracommunity density is


(11) $$\theta_{\mathrm{intra}}^{\left(c\right)}=\frac{1}{k_{c}}\sum_{i\in c}p_{i},$$

and $\theta_{\mathrm{intra}}^{\left(c\right)}$ decreases (linearly or nonlinearly) as the community index *c* grows. The intercommunity density between adjacent communities *c* and c+1 scales with the smaller of the two,


(12) $$\theta_{\mathrm{inter}}^{\left(c,c+1\right)}={\left(\theta_{\mathrm{intra}}^{\left(c+1\right)}\right)}^{2},$$

producing networks with both highly dense and very sparse communities, a regime where single-level cuts cannot resolve the structure.

### State space

The MCMC state space is the set of all valid adaptive cuts of the dendrogram; equivalently, sets of internal nodes whose subtrees partition all leaves. The number of valid cuts of a binary dendrogram rooted at *v* is given by the recursion


(13) $$a(v)=\left\{ \begin{array}{cc} 1 & \text{if }v\text{ is a leaf, }\\ 1+a(\ell )\cdot a(r) & \text{otherwise}, \end{array}\right.$$

where ℓ and *r* are the children of *v* and the leading 1 counts the trivial cut {v}. For a perfectly balanced binary tree on $n=2^{d}$ leaves this gives $a_{d}=1+a_{d-1}^{2}$ (OEIS A003095), which grows doubly exponentially in depth *d*, giving $a_{d}=2^{\Theta (n)}$ (single-exponential in *n*, since $\log_{2}\;a_{d}\approx 0.59n$). This is dramatically smaller than the Bell number B(n) that counts all partitions of *n* elements, and is what makes the adaptive-cut MCMC tractable.

### Reversible-jump MCMC sampler

Because moves between adaptive cuts change the number of clusters, the sampler is a reversible-jump MCMC (RJMCMC) (29). Given a partition with *k* clusters, each proposal step proceeds as follows.

1. Select a cluster $v_{i}$ uniformly at random with probability 1/k.
2. Choose a direction: forced up ($P_{\mathrm{up}}=1$) if $v_{i}$ is a dendrogram leaf, forced down ($P_{\mathrm{down}}=1$) if $v_{i}$ is the root, otherwise $P_{\mathrm{up}}=P_{\mathrm{down}}=1/2$.
3. Execute the move. Up: merge $v_{i}$ with its dendrogram sibling into their parent; if the sibling has been split into smaller descendants, collapse all of them. Down: sample a random adaptive cut *σ* of $v_{i}$’s subtree from the geometric-penalty proposal
  (14) $$\rho (\sigma |v_{i})=\frac{2^{-|\sigma |}}{Z_{c}(v_{i})},$$
  where the constrained partition function $Z_{c}(v_{i})$ restricts *σ* so that at least one direct child of $v_{i}$ remains whole. This guarantees that the reverse of any down-move is an up-move from a whole direct child; when both direct children remain whole, either can trigger the reverse and the reverse proposal sums their contributions (Eq. 17).

The normalization constants are precomputed bottom-up in O(n):


(15) $$\begin{array}{rl} Z(v) & =\left\{ \begin{array}{cc} 1/2 & \text{if }v\text{ is a leaf, }\\ 1/2+Z(\ell )\;Z(r) & \text{otherwise, } \end{array}\right. \end{array}$$

(16) $$Z_{c}(v)=\frac{1}{2}Z(\ell )+\frac{1}{2}Z(r)-\frac{1}{4}.$$

Values are stored in log-space for large subtrees.

The resulting proposal probability $Q_{x\to y}$ for a transition from state *x* (with *k* clusters) to state *y* is asymmetric:


(17) $$Q_{x\to y}=\left\{ \begin{array}{cc} \frac{1}{k}\sum_{v\in S_{\mathrm{up}}}P_{\mathrm{up}}(v), & \text{if }y\text{ is an up-move from }x,\\ \frac{1}{k}\;P_{\mathrm{down}}(v_{i})\;\rho (\sigma |v_{i}), & \text{if }y\text{ is a down-move from }x,\\ 0 & \text{otherwise}, \end{array}\right.$$

where $S_{\mathrm{up}}$ is the set of clusters in *x* whose selection and up-move yields *y*. Although *Q* is not symmetric, the Metropolis–Hastings rule (40, 41) preserves detailed balance for any ergodic proposal by incorporating the proposal-density ratio. The acceptance probability is


(18) $$\alpha (x_{i}\to x_{⋆})=\min\left\{1,\frac{\pi^{⋆}(x_{⋆})Q_{x_{⋆}\to x_{i}}}{\pi^{⋆}(x_{i})\;Q_{x_{i}\to x_{⋆}}}\right\}=\min\left\{1,e^{\Delta f/T}\cdot \frac{Q_{x_{⋆}\to x_{i}}}{Q_{x_{i}\to x_{⋆}}}\right\},$$

where $\Delta f=f(x_{⋆})-f(x_{i})$. With this acceptance probability the stationary distribution is $\pi^{⋆}(x)\propto e^{\;f(x)/T}$ on the space of adaptive cuts. The matched-support property ($Q_{x\to y}>0\;⟺\;Q_{y\to x}>0$), detailed balance, and ergodicity are proved formally in Section S2.

To prevent the chain from getting trapped in local maxima we use the logarithmic simulated-annealing (42) cooling schedule


(19) $$T_{k}=\frac{T_{0}}{\log\;(k-k_{\mathrm{SA}}+2)},\;k>k_{\mathrm{SA}},$$

with $T_{k}=T_{0}$ for $k\leq k_{\mathrm{SA}}$. We use $k_{\mathrm{SA}}=2N_{\mathrm{steps}}/3$ by default, which lets the chain explore at high temperature before converging.

### Balancedness axioms

The balancedness score *B* (Eq. 5) satisfies the three universal tree-balance axioms of (24).

#### Maximum-value axiom

For every level *l*, $H_{\min}(\pi_{l})\leq H(\pi_{l})\leq H_{\max}(\pi_{l})$, so each summand of *B* lies in [0,1] and 0≤B≤1. Equality B=1 requires $H(\pi_{l})=H_{\max}(\pi_{l})$ at every level, i.e. every internal node splits its leaves into branches of identical size.

#### Minimum-value axiom

In a caterpillar (fully unbalanced) dendrogram every internal node separates a single leaf from the rest, so $H(\pi_{l})=H_{\min}(\pi_{l})$ at every level and B=0. Conversely, B=0 implies every split ejects a single leaf, i.e. the dendrogram is a caterpillar.

#### Insensitivity to vanishing subtrees

Replacing a leaf by a subtree with εn leaves (ε→0) perturbs each entropy by O(εlog(1/ε)). Since the denominators $H_{\max}-H_{\min}$ are independent of ε, each summand of *B* perturbs by at most Cεlog(1/ε), so $|B(T_{\varepsilon })-B(T)|\to 0$.

### Computational complexity

The sampler performs a one-off O(M) precomputation of the geometric-cut normalizers $\log\;Z(v),\log\;Z_{c}(v)$, the per-node leaf sets, the down-move probability tables, and the per-subtree partition-density contributions $D(v)=m_{v}D_{c}(m_{v},n_{v})$. The per-step cost of the main loop is then O(k) for an up-move (where k=|σ| is the current number of communities and the dominant cost is a sibling-leaf disjointness scan for the multicollapse) and $O(|\sigma^{\prime }|)$ for a down-move that samples a cut of size $\left|\sigma^{\prime }\right|$ from $\rho (\sigma |v)=2^{-|\sigma |}/Z_{c}(v)$. Accepting a move updates the total partition density incrementally from the cached D(v) values in $O(|\sigma^{\prime }-\sigma |)$. The total optimization cost is $O(M)+O(N_{\mathrm{steps}}\cdot \bar{c})$, where $\bar{c}$ is the average move cost; in practice $\bar{c}$ stays small because the chain spends most of its time near a low-*k* partition.

We benchmarked the sampler at $2\times 10^{5}$ steps, $T=10^{-4}$, on a single CPU process across ten networks spanning 78≤M≤18,083 edges (Table S6). Wall-clock spans 1–10s for M<1,000 and tens of seconds to a few minutes for 1,000≤M<10,000, with the high-improvement networks at the slow end (e.g. bible_nouns, M=9,059, 184s); networks with M≥10,000 take 1–10min. Dendrogram construction (Jaccard similarities and single-linkage) overtakes the MCMC only for the largest networks (e.g. us_congress-H93, M=18,083). Convergence, initialization-robustness, temperature-sensitivity, and reproducibility diagnostics are reported in Section S5. The full catalog of networks used to evaluate the method (with balancedness, $D_{\mathrm{lc}}$, $D_{\mathrm{ac}}$, and provenance) is provided as Table S11 (Section S7).

## Discussion

In this article, we introduced the adaptive cut, a new method designed to overcome the limitations of traditional single-level cuts in hierarchical clustering and community detection. Using multilevel cuts and optimizing an objective function using a Markov chain Monte Carlo algorithm with simulated annealing, the adaptive cut uses the full hierarchical structure of dendrograms. This approach is best suited to datasets with unbalanced dendrograms, where single-level cuts often fail to capture the true underlying structure. We also introduce the balancedness score, an information-theoretic metric that quantifies the balance of a dendrogram and predicts the potential benefits of the adaptive cut.

First, we demonstrated that traditional single-level cuts can be inadequate for accurately identifying clusters in complex networks, especially when dealing with unbalanced dendrograms, which can arise when the density of data is uneven across a dataset. Single-level cuts may lead to over-aggregation in some parts of the dendrogram and overly granular partitions in others, resulting in suboptimal clustering. This issue was evident in our experiments with SBMs exhibiting varying densities, where the adaptive cut outperformed the single-level approach.

Second, we introduced the balancedness score, an information-theoretic metric that quantifies the balance of a dendrogram. Our analysis of over 200 real-world networks revealed that most dendrograms are unbalanced. The balancedness score not only measures dendrogram balance but also serves as a predictor of the potential improvement achievable with the adaptive cut method. Networks with lower balancedness scores tended to benefit more from the adaptive cut, as it adjusts for the imbalance by employing multilevel cuts.

Applied to both synthetic and real-world networks, the adaptive cut outperforms single-level cuts. In synthetic networks, the adaptive cut produced partitions that more accurately reflected the ground truth community structures, especially in models with varying densities. In real-world networks across diverse domains (including social, economic, biological, and transportation networks), the adaptive cut improved performance metrics such as partition density and modularity. These improvements were most pronounced in networks with unbalanced dendrograms.

We showed the generality of the adaptive cut method. It can be applied across different hierarchical clustering techniques (e.g. single linkage, average linkage, Ward’s method) and optimized for objective functions such as modularity, silhouette score, and partition density. This flexibility lets the adaptive cut apply to many clustering tasks beyond network analysis, including machine learning tasks that use hierarchical clustering.

We distinguish adaptive cut from other multiscale community detection approaches. Methods based on multiresolution modularity (43) scan a resolution parameter to identify communities at different scales, but operate on the full partition space rather than leveraging dendrogram structure. Stability-based methods (44) analyze community persistence across Markov time scales, requiring diffusion processes rather than an existing dendrogram. Dynamic tree cut (20) is the most closely related approach, as it also produces multilevel cuts of dendrograms, but relies on shape-based heuristics rather than objective function optimization. The key distinction is that adaptive cut is not a standalone community detection method but a complementary postprocessing step: it takes any given dendrogram as input and optimizes the cut, regardless of how the dendrogram was constructed.

The balancedness index, beyond its role as an improvement predictor, serves as a structural descriptor of community heterogeneity. The quantity *B* reads the spread of merge heights in the dendrogram: a dendrogram whose merges are concentrated near a single similarity gives *B* near 1, while a dendrogram with widely spread merge heights gives *B* near 0. A controlled 2×2 design that crosses uniform vs. varying intracommunity density against flat vs. nested community structure (Section S6.3) shows that the spread is driven by density heterogeneity across communities, not by the depth of nesting: *B* drops by 0.09–0.12 along the density-variation axis and changes by <0.04 along the nesting axis. The density-axis ordering also holds under average and complete linkage; under single linkage on larger graphs the chaining effect compresses *B* into a narrow band, so both the absolute scale of *B* and the resolvability of the density signal depend on the linkage choice (Fig. S8). Balancedness is related to, but distinct from, multiscale notions arising from the resolution limit of modularity optimization (31) and from multiresolution (43) and Markov-stability (44) community-detection schemes, which target community size relative to total network scale. Density heterogeneity is one ingredient of multiscale structure: low *B* identifies networks where some communities are tight and others are loose, the regime in which a single-level cut over- or underfragments and where adaptive cut helps most.

While the adaptive cut offers advantages, it has limitations. One key limitation is its reliance on the dendrogram structure, which confines the set of possible partitions to those represented within the dendrogram. Although this restriction reduces the state space and enhances computational efficiency, it may exclude some potentially optimal partitions not present in the dendrogram.

The optimization process within the adaptive cut uses a Markov chain Monte Carlo method with a simulated annealing schedule. The choice of cooling schedule can impact convergence rates and computational efficiency. Future work could investigate adaptive cooling schemes, where the cooling rate is adjusted dynamically based on the acceptance rate of new solutions. This approach could provide a more flexible optimization strategy, enhancing convergence and allowing the algorithm to balance exploration and exploitation.

Another promising direction is to explore alternative optimization methods, such as dynamic programming, which can optimize objective functions on tree structures with lower computational complexity (45, 46).

In conclusion, the adaptive cut method, coupled with the balancedness score, represents an advance in hierarchical clustering and community detection. By using the hierarchical information in dendrograms and addressing the shortcomings of single-level cuts, it provides a more accurate clustering method suitable for complex and unbalanced datasets.

## Supplementary Material

pgag344_Supplementary_Data

## Data Availability

The code and data underlying this article are available at github.com/LCB0B/adaptive_cut.

## Preprints

This manuscript was posted on a preprint: https://arxiv.org/abs/2512.08741.
