# Supplementary material for: Adaptive cut reveals multiscale complexity in networks
Source: PNAS Nexus. 2026 Oct 6;5(11):pgag344. doi: 10.1093/pnasnexus/pgag344 (PMC13741240; doi:10.1093/pnasnexus/pgag344)
Supplement: Supplementary file 1 [file pgag344_supplementary_data.pdf]

Supplementary Information

Adaptive cut reveals multiscale complexity in networks

Louis Boucherie, Yong-Yeol Ahn, Sune Lehmann

Contents

S1 Code availability 2

S2 Reversible-Jump MCMC: Detailed Balance and Ergodicity 2
- S2.1 Sampler correctness . . . 2
- S2.2 Matched support of proposals (UP $\leftrightarrow$ DOWN pairing) . . . 3
- S2.3 Detailed balance and ergodicity . . . 3

S3 Greedy Baseline 4

S4 Adaptive Cut Across Linkage Methods 6

S5 Convergence and Sensitivity Diagnostics 7
- S5.1 Convergence vs. chain length . . . 7
- S5.2 Robustness to initialisation . . . 8
- S5.3 Temperature sensitivity . . . 9
- S5.4 Complexity and wall-clock timing . . . 10

S6 Balancedness as a Structural Descriptor 10
- S6.1 Decision criterion . . . 10
- S6.2 Partition disagreement, tree balance, and multiscale span . . . 11
- S6.3 What B captures: a controlled 2×2 design . . . 12
- S6.4 Relation to the Fernández–Gómez normalised tree balance . . . 13

S7 Network Catalogue 14

S8 Adaptive Cut on the Louvain Dendrogram 19

S1 Code availability

Code is available on GitHub `https://github.com/LCB0B/adaptive_cut`.

S2 Reversible-Jump MCMC: Detailed Balance and Ergodicity

The adaptive-cut sampler defined in Methods is a reversible-jump MCMC [1] on the space $\mathcal{X}$ of adaptive cuts of a binary dendrogram. Every transition is either a single-cluster up-move (collapse the selected cluster with its sibling, possibly absorbing the sibling's descendants) or a down-move that samples a constrained adaptive cut of the selected cluster's subtree from the geometric-penalty proposal $\rho(\sigma \mid v) = 2^{-|\sigma|}/Z_c(v)$ (Eq. 14 of Methods). In this section we (i) verify that the unconstrained and constrained proposals are bona-fide probability distributions over cuts, (ii) prove that up- and down-moves come in reverse pairs with $Q_{x\to y} > 0 \iff Q_{y\to x} > 0$ (matched support), and (iii) conclude that the Metropolis–Hastings acceptance rule preserves the target $\pi^\star(x) \propto e^{f(x)/T}$ and produces an ergodic chain. Throughout, $\Sigma(v)$ denotes the set of adaptive cuts of the subtree rooted at v and $\Sigma_c(v) \subset \Sigma(v)$ the constrained cuts in which at least one direct child of v remains whole, so that the state space is $\mathcal{X} = \Sigma(\text{root})$.

S2.1 Sampler correctness

Lemma S2.1 (Single-cut proposal normalises)**.** *For every node v of the dendrogram, the unconstrained geometric-penalty proposal $\rho_u(\sigma \mid v) = 2^{-|\sigma|}/Z(v)$ defines a probability distribution over the set of adaptive cuts of the subtree rooted at v, with*

$$Z(v) = \begin{cases} 1/2 & \text{if } v \text{ is a leaf,} \\ 1/2 + Z(\ell)\, Z(r) & \text{if } v \text{ has children } \ell, r. \end{cases} \tag{S2.1}$$

Proof. By structural induction on v. If v is a leaf, the only cut is $\{v\}$ with $|\sigma| = 1$, so $\sum_\sigma 2^{-|\sigma|} = 1/2 = Z(v)$. If v has children ℓ, r, every cut either equals $\{v\}$ (contributing $2^{-1} = 1/2$) or descends past v, in which case it decomposes uniquely as $\sigma = \sigma_\ell \sqcup \sigma_r$ with $\sigma_\ell \in \Sigma(\ell)$, $\sigma_r \in \Sigma(r)$ and $|\sigma| = |\sigma_\ell| + |\sigma_r|$: every block of an adaptive cut is a subtree root, so no block straddles the two children, and $\sigma \mapsto (\sigma_\ell, \sigma_r)$ is a bijection onto $\Sigma(\ell) \times \Sigma(r)$. These cuts contribute $\sum_{\sigma_\ell} 2^{-|\sigma_\ell|} \cdot \sum_{\sigma_r} 2^{-|\sigma_r|} = Z(\ell)\, Z(r)$ by the inductive hypothesis. Summing gives $Z(v) = 1/2 + Z(\ell)\, Z(r)$. □

Lemma S2.2 (Constrained-cut proposal normalises)**.** *The constrained proposal $\rho_c(\sigma \mid v) = 2^{-|\sigma|}/Z_c(v)$ over adaptive cuts σ of v's subtree in which at least one direct child of v remains whole is a probability distribution, with*

$$Z_c(v) = \tfrac{1}{2} Z(\ell) + \tfrac{1}{2} Z(r) - \tfrac{1}{4}. \tag{S2.2}$$

Proof. Let A be the adaptive cuts of v's subtree in which ℓ remains whole and B those in which r remains whole; the constrained set is $A \cup B$. Summing the geometric weight over A gives $\sum_{\sigma_r} 2^{-1-|\sigma_r|} = Z(r)/2$ (with ℓ whole, the rest of the cut is any cut of r's subtree), and over B gives $Z(\ell)/2$. The overlap $A \cap B$ contains only $\sigma = \{\ell, r\}$: once both children are whole they already cover every leaf of v, so no further node can be added without re-covering a leaf or re-introducing v; its weight is $2^{-2} = 1/4$. Inclusion–exclusion yields $Z_c(v) = Z(\ell)/2 + Z(r)/2 - 1/4$. □

Lemma S2.3 (Bottom-up linear computability)**.** *Both $Z(v)$ and $Z_c(v)$ for every node v can be computed in $\mathcal{O}(n)$ time and stored in log-space, where n is the number of leaves of the dendrogram (the number of edges M in the link-clustering application).*

Proof. A single post-order traversal computes $Z(v)$ from its children using Eq. S2.1; a second post-order traversal computes $Z_c(v)$ using Eq. S2.2. Each node is visited a constant number of times. □

S2.2 Matched support of proposals (UP ↔ DOWN pairing)

Lemma S2.4 (UP → DOWN pairing)**.** *Let y be the state obtained from x by an up-move at cluster $v_i \in x$. Then $Q_{x\to y} > 0$ and there exists a unique reverse down-move from y to x, with $Q_{y\to x} > 0$.*

Proof. Let p be the parent of v_i in the dendrogram and let v_s be the dendrogram sibling of v_i. The up-move removes from x the cluster v_i together with every cluster of x that is a descendant of v_s (a set $R \subseteq x$), and adds the cluster p, producing y. Since x is a cut, the clusters of x lying under v_s partition v_s's leaves, so together with $\{v_i\}$ (which covers v_i's subtree) the set R covers p's subtree exactly once, i.e. $R \in \Sigma(p)$. Two cases arise. If $v_s \in x$ (the sibling is whole), then $R = \{v_i, v_s\}$ and the same y is reached by selecting either v_i or v_s, so $S_{\mathrm{up}} = \{v_i, v_s\}$. If $v_s \notin x$ (the sibling has been split), then $|R| \geq 3$ (covering the split subtree of v_s needs at least two clusters, plus v_i) and only the selection of v_i yields y, so $S_{\mathrm{up}} = \{v_i\}$. In both cases $Q_{x\to y} = (1/|x|) \sum_{v\in S_{\mathrm{up}}} P_{\mathrm{up}}(v) > 0$. For the reverse, v_i is a direct child of p by construction; selecting p in y (probability $1/|y|$) and proposing the constrained cut $\sigma = R$ of p's subtree, in which the direct child v_i of p remains whole ($v_i \in R$, so $R \in \Sigma_c(p)$), inverts the up-move. Its probability is $Q_{y\to x} = (1/|y|)\, P_{\mathrm{down}}(p)\, 2^{-|R|}/Z_c(p) > 0$. □

Lemma S2.5 (DOWN → UP pairing)**.** *Let y be the state obtained from x by a down-move at cluster $v_i \in x$ producing a constrained cut σ. Then $Q_{x\to y} > 0$, and the reverse transition $y \to x$ has $Q_{y\to x} > 0$, triggered by every whole direct child of v_i in σ.*

Proof. Since the only size-one cut of v_i's subtree is $\{v_i\}$ itself, which keeps no direct child whole, every $\sigma \in \Sigma_c(v_i)$ has $|\sigma| \geq 2$, so a down-move strictly increases the cluster count and $y \neq x$. Because σ is a constrained cut, the set W of direct children of v_i that are whole in σ is non-empty. For each $w \in W$, selecting w in y (probability $1/|y|$) and proposing an up-move collapses w together with the clusters of y in the subtree of w's sibling (the sibling itself if whole, or its descendants if split; in either case exactly $\sigma \setminus \{w\}$) and restores v_i. Conversely, any one-step move from y that yields x must increase the cluster count, so it is an up-move, and must reinsert v_i, which forces the selected cluster to be a direct child of v_i present in σ, i.e. an element of W. The reverse selections are exactly W, and $Q_{y\to x} = (1/|y|) \sum_{w\in W} P_{\mathrm{up}}(w) > 0$. When only one child is whole, $|W| = 1$; when both children are whole, $|W| = 2$ and the two contributions add. □

S2.3 Detailed balance and ergodicity

Theorem S2.6 (Detailed balance)**.** *For the proposal Q defined in Eq. 17 of Methods and the Metropolis–Hastings acceptance probability $\alpha(x \to y) = \min\{1, \pi^\star(y)Q_{y\to x}/[\pi^\star(x)Q_{x\to y}]\}$ (Eq. 18 of Methods), the resulting transition kernel $P(x \to y) = Q_{x\to y}\, \alpha(x \to y)$ (for $y \neq x$) satisfies*

$$\pi^\star(x)\, P(x \to y) = \pi^\star(y)\, P(y \to x) \qquad \textit{for all } x, y \in \mathcal{X}. \tag{S2.3}$$

Proof. An up-move strictly decreases the cluster count (it removes $|R| \geq 2$ clusters and adds their parent) while a down-move strictly increases it (it removes one cluster and adds $|\sigma| \geq 2$), so for any ordered pair (x, y) at most one case of Eq. 17 of Methods applies and $Q_{x\to y}$ is single-valued. Lemmas S2.4 and S2.5 show that $Q_{x\to y} > 0 \iff Q_{y\to x} > 0$ for every pair $(x, y) \in \mathcal{X}^2$. For such a

pair,

$$\pi^\star(x)\,Q_{x\to y}\,\alpha(x\to y) = \min\{\pi^\star(x)\,Q_{x\to y},\ \pi^\star(y)\,Q_{y\to x}\}$$
$$= \pi^\star(y)\,Q_{y\to x}\,\alpha(y\to x),$$

by the symmetry of the min. For pairs with $Q_{x\to y} = Q_{y\to x} = 0$, both sides of Eq. S2.3 vanish. □

Corollary S2.7 (Metropolis–Hastings applicability)**.** *The chain defined by Eqs. 17 and 18 of Methods satisfies the sufficient conditions for a convergent Metropolis–Hastings sampler. (i)* Irreducibility. *From any state $x \in \mathcal{X}$, repeated up-moves reach the root state $\{r\}$ in at most $|x|-1$ steps, since each up-move removes at least two clusters and adds one, strictly decreasing $|x|$. From $\{r\}$, any target y is reached by at most $|y|-1$ down-moves: whenever a current cluster v strictly contains more than one cluster of y, propose the minimal constrained cut consisting of the two direct children of v (both whole, always in $\Sigma_c(v)$, with probability $1/[4Z_c(v)] > 0$), which refines the partition by one cluster; iterating reaches y, so $P^{(N)}(x,y) > 0$ for some $N \le |x| + |y| - 2 \le 2(M-1)$. (ii)* Matched support*: $Q_{x\to y} > 0 \iff Q_{y\to x} > 0$ by Lemmas S2.4–S2.5. (iii)* Evaluable positive target*: $\pi^\star(x) = e^{f(x)/T}/Z > 0$ enters α only through the ratio $\pi^\star(y)/\pi^\star(x) = e^{(f(y)-f(x))/T}$, so the normaliser Z is never needed. (iv)* Detailed balance *holds by Theorem S2.6. (v)* Aperiodicity*: the proposal is asymmetric and does not by itself satisfy detailed balance with respect to $\pi^\star$, so some move is accepted with probability $\alpha < 1$; the corresponding rejection gives a positive self-transition probability $P(x \to x) > 0$, so x has period 1 and, with irreducibility, the chain is aperiodic. By (i)–(v) and the ergodic theorem for finite irreducible aperiodic Markov chains [2], $\pi^\star$ is the unique stationary distribution and $P^n(x,\cdot) \to \pi^\star$ in total variation from every initial state.*

These guarantees hold at each fixed temperature T, where the kernel has stationary law $\pi^\star_T \propto e^{f/T}$. As $T \to 0$ the law $\pi^\star_T$ concentrates on the maximisers of f, but the time-inhomogeneous chain run under the cooling schedule T_k (Eq. 19 of Methods) inherits this concentration only under a sufficiently slow schedule. For $T_k = c/\log k$, Hajek's theorem [3] gives convergence in probability to a global maximiser of f exactly when $c \ge d^\star$, where $d^\star$ is the largest depth (measured in f) of a basin surrounding a non-global local maximum. The deployed numerator T_0 is small (of order $0.1/M$) and likely below $d^\star$, so we use the schedule as a simulated-annealing heuristic that biases the chain toward high-f states rather than as a proof of global optimality; convergence behaviour is assessed empirically in Section S5.

S3 Greedy Baseline

To isolate the contribution of the stochastic, multi-level Metropolis–Hastings sampler from the multi-cut principle itself, we compared the adaptive cut against a deterministic greedy baseline operating on the same single-linkage dendrogram and optimising the same objective (D). Starting from σ_{lc}, the greedy procedure scans the current communities and applies any UP move (merge with dendrogram sibling, absorbing descendants when needed) or DOWN move (split into a sampled adaptive cut of the subtree) that strictly increases D, iterating until no improving move is found. The greedy baseline shares the move set of the RJMCMC sampler but removes all stochasticity and all sub-optimal acceptances.

We ran the greedy baseline on all 202 networks in the catalogue (Section S7). Greedy alone improves over σ_{lc} on 156/202 networks (77%, mean improvement +21%, median +12%), with the largest gains on highly unbalanced dendrograms (`Erdos` +135.7%, `eu_procurements_alt-AT_2008` +131.1%, `STEM concept networks Researchers` +126.0%, `foursquare-NYC_restaurant_checkin`

+83.0%, `bitcoin_alpha` +70.1%). The multi-cut principle, separated from the MCMC, already produces large gains over the single-level cut.

The full RJMCMC sampler matches or exceeds the greedy baseline on every network. Across all 202 networks, $D_{\mathrm{ac}} \geq D_{\mathrm{greedy}}$ with zero violations; $D_{\mathrm{ac}} > D_{\mathrm{greedy}}$ strictly on 196/202 networks (97%). The RJMCMC mean improvement is +34% (+21% median), which leaves a $\sim$ 13-percentage-point mean gap between RJMCMC and greedy. This gap quantifies the contribution of the geometric-penalty multi-level DOWN proposal (Eq. 14 of Methods) combined with simulated annealing: the chain reaches dendrogram-consistent multi-level cuts that the deterministic greedy cannot escape to from a local optimum.

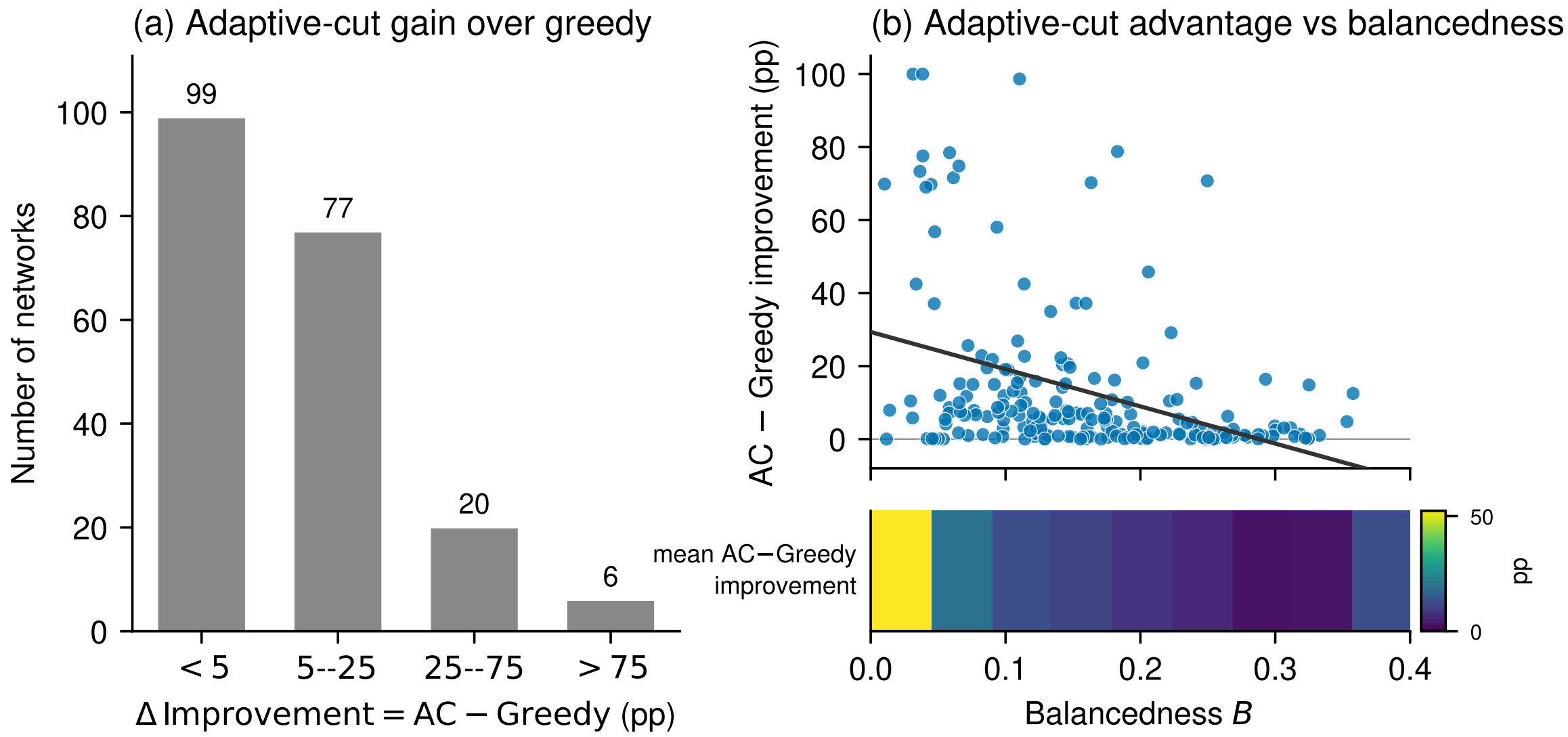

Figure S1: Greedy baseline vs adaptive-cut RJMCMC, both operating on the same single-linkage dendrogram and optimising partition density D, on all 202 networks in the catalogue (Section S7). **(a)** Distribution of the per-network gain of the adaptive cut over greedy, Δ Improvement $=$ Improv.$_{\mathrm{AC}}$ $-$ Improv.$_{\mathrm{greedy}}$, in percentage points of improvement over the single-level cut D_{lc}, bucketed as $<$ 5, 5–25, 25–75, and $>$ 75 pp. Greedy improves on 156/202 networks (mean +21%, median +12%); the full RJMCMC sampler improves on 195/202 (mean +34%, median +21%) and matches or exceeds greedy on every network. **(b)** The same advantage AC $-$ Greedy as a function of dendrogram balancedness B. Top: one point per network with a linear fit (Pearson $r = -0.37$); two networks above 100 pp are shown at the cap. Bottom: the mean advantage in each B bin as a heat strip. The adaptive cut's gain over greedy concentrates at low B, where communities are spread across dendrogram levels and the deterministic greedy is trapped in a local optimum.

Panel (a): the adaptive cut's gain over greedy exceeds 5 pp on 103/202 networks, with 6 of them above 75 pp. Panel (b): this gain concentrates at low balancedness (Pearson $r = -0.37$), the regime where multiscale structure is spread across dendrogram levels and the deterministic greedy cannot escape its local optimum.

This separates three effects: (i) the multi-cut principle itself accounts for about 21 of the 34 percentage points of mean gain over σ_{lc} and is recoverable by a deterministic algorithm; (ii) the RJMCMC's specific design (geometric-penalty multi-level proposal $\rho(\sigma \mid v) = 2^{-|\sigma|}/Z_c(v)$ plus simulated annealing) adds the remaining $\sim$ 13 percentage points by escaping greedy local optima; (iii) the exploration is not a generic resolution sweep, since both methods stay within the family of

dendrogram-consistent cuts.

Dynamic Tree Cut comparison. As a published-baseline check we also ran Langfelder et al.'s `dynamicTreeCut` [4] on 30 catalogue networks spanning $M = 23$ to 44,000 and $B \in [0.034, 0.358]$, sweeping the hyperparameter grid `deepSplit` $\in \{0, \ldots, 4\}$, `minClusterSize` $\in \{2, 5, 10, M/1000\}$, and both standard handlings of unassigned leaves. The best DTC partition per network gives a mean change of -41.5% relative to $D_{\rm lc}$ (median -45.1%) and never beats $\sigma_{\rm lc}$ on any of the 30 networks; adaptive cut delivers a higher D than DTC on all 30 (`results/dtc_comparison_30.csv`). This is expected: DTC's selection rules are objective-agnostic, using branch-shape statistics rather than partition density, and since $\sigma_{\rm lc}$ is by construction the D-optimal horizontal cut, any D-agnostic horizontal-cut rule is upper-bounded by $D_{\rm lc}$. AC beating DTC on D is forced; the comparison shows instead that adaptive cut combines DTC's multi-level idea with objective-aware optimization. The greedy baseline above is the cleaner ablation since it shares both the move set and the objective D with adaptive cut.

S4 Adaptive Cut Across Linkage Methods

To verify that the adaptive cut is agnostic to the dendrogram-construction method, we repeat the link-clustering experiment using four standard linkages: single, average (UPGMA), weighted (WPGMA), and Ward.

For each network and each linkage method, we (i) compute the link similarity matrix as in the main text (Methods, Eq. 8), (ii) build a dendrogram using the corresponding linkage criterion, (iii) compute the optimal single-level cut by maximising partition density D along the dendrogram, and (iv) run the adaptive cut MCMC initialised at the single-level optimum. We report the partition density of the single-level cut $D_{\rm lc}$, the partition density of the adaptive cut $D_{\rm ac}$, the relative improvement, and the balancedness B of the dendrogram.

We evaluate twenty networks with $78 \leq M \leq 1{,}954$ links spanning social, biological, technological, transportation, and informational domains, drawn from our benchmark catalogue (Section S7). For each network we build the dendrogram with each of four linkage methods (single, average, weighted, Ward) applied to the same Jaccard edge-similarity matrix, then run the adaptive cut on each dendrogram. Table S1 reports the results across all eighty network–linkage combinations.

Two patterns emerge. First, average, weighted, and Ward linkage produce more balanced dendrograms than single linkage. Across the twenty networks, single linkage yields balancedness scores in the range $B = 0.08$–0.33, whereas the other three methods yield $B = 0.26$–0.75. This is consistent with the chaining behaviour of single linkage, which tends to absorb leaves one at a time into a growing cluster and produce caterpillar-like trees.

Second, the adaptive cut improves partition density on most network–linkage combinations. Single-linkage gains span $+2.4\%$ to $+142\%$ with a median of $+18\%$. Gains for the other three linkages span -85% to $+77.8\%$, where the two negative cases (average and weighted linkage on eu-proc-AT) are near-degenerate runs with $D_{\rm lc} \approx 0.001$–0.002 and the chain drifts off the single-level optimum within a single annealing window. As predicted by the balancedness analysis (Fig. 4), the largest single-linkage gains are concentrated in the more unbalanced dendrograms, but meaningful improvements persist on the more balanced trees produced by average, weighted, and Ward linkage.

The adaptive cut is agnostic to the choice of linkage method: it improves partition density across all four methods on the bulk of the test set, and the magnitude of the improvement is well predicted by the balancedness score of the underlying dendrogram. They also show that, on the

part of the catalogue where single linkage is competitive on the adaptive-cut objective, the gain from the adaptive cut is largest for single linkage.

S5 Convergence and Sensitivity Diagnostics

This section quantifies how the adaptive-cut MCMC behaves as a function of chain length, initialisation, and temperature, and gives a complexity analysis together with a wall-clock benchmark. The supporting scripts and result files are listed at the end of each subsection.

S5.1 Convergence vs. chain length

Figure S2 shows fractional improvement $(D - D_{\mathrm{lc}})/D_{\mathrm{lc}}$ as a function of MCMC step for two complementary experiments. Panel (a) plots 25 networks in the moderate-improvement band (5–25% gain), each at the heuristic temperature with two independent seeds, logged every 1,000 steps for 5×10^5 steps. Panel (b) plots five reference networks (Zachary, contiguous_usa, foodweb_baywet, cintestinalis, euroroad) at a step-grid (10^2 to 10^5).

Two observations:

- For 22 of the 25 networks in panel (a), the trajectory at the heuristic temperature is within $|\Delta D| < 0.003$ of its running maximum by 2×10^5 steps, and within $|\Delta D| < 0.001$ by 4×10^5 steps; the remaining three networks drift inside a band of width < 0.013. The chain has mixed below our default 2×10^5-step budget on this band of networks.
- Panel (b) confirms that for networks with $M < 5{,}000$ edges, 10^4 steps is sufficient: `Zachary` and `contiguous_usa` reach their plateau by 1,000 steps; `foodweb_baywet` and `cintestinalis` by 5,000 steps; `euroroad` still creeps slightly between 10^4 and 10^5 steps (within $\sim 4\%$ of its 10^5-step value at 10^4 steps), the only network in this set that benefits from chains longer than 10^4 steps.

Table S2 reports D at three step counts on a grid for the five reference networks of panel (b).

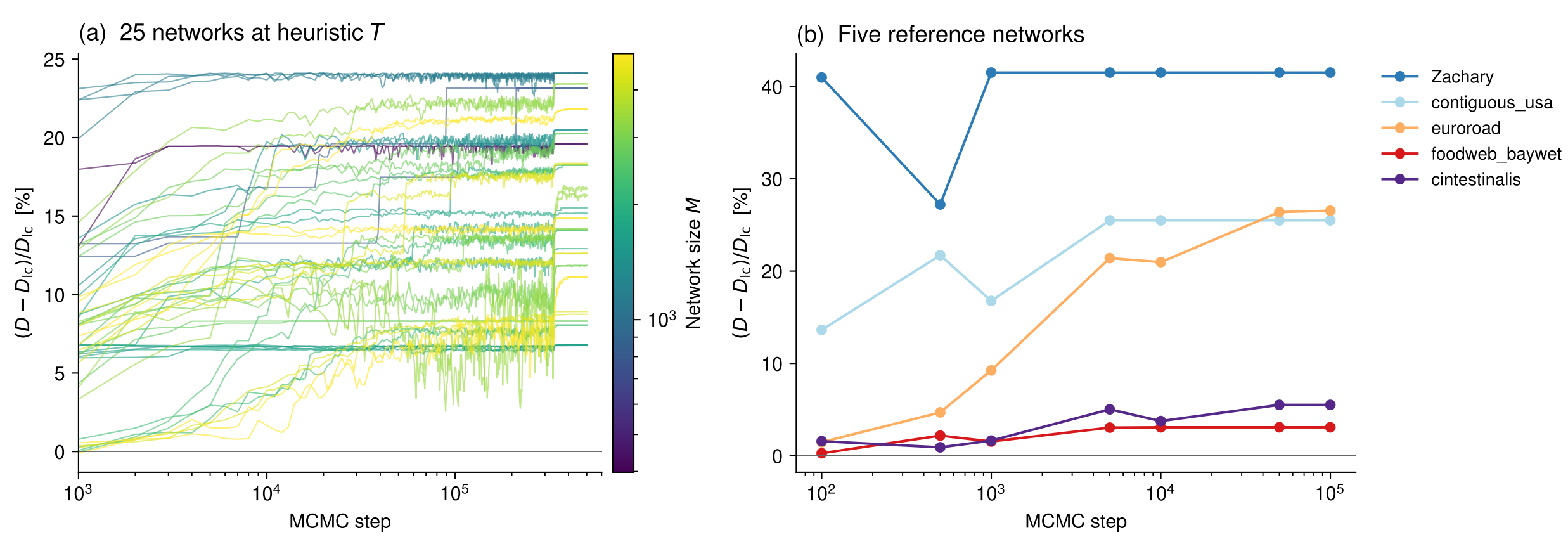

Figure S2: Convergence of D along MCMC chains. (a) Fractional improvement vs. step for 25 networks in the 5–25% improvement band at the heuristic temperature, two trials per network. (b) Five reference networks on a step grid spanning 10^2 to 10^5 steps. All but `euroroad` reach their 10^5-step value at 10^4 steps; `euroroad` is within $\sim 4\%$ at 10^4 steps.

S5.2 Robustness to initialisation

The MCMC is initialised at the single-level cut σ_{lc} in all results of the main text and Supplementary Material. This initialisation is justified because σ_{lc} is the global maximum of D along single-level cuts of the dendrogram, a well-defined high-density starting point in the multi-level cut space.

To check that the final D_{mcmc} is not an artefact of this initialisation, we ran the chain from three starting partitions ($k_{\text{start}} \in \{k_{\text{lc}}, M/10, M\}$) on a fixed eight-network set (Zachary, Les Miserables, Football, euroroad, foodweb_baywet, cintestinalis, Jazz, bible_nouns; the same eight are reused for the temperature sweep in Section S5.3), for 5×10^4 MCMC steps at the heuristic T_0 (Fig. S3). Five of the eight networks (`Zachary`, `Les Miserables`, `euroroad`, `foodweb_baywet`, `Jazz`) reach the same plateau from all three initialisations; the chain initialised at σ_{lc} converges first because σ_{lc} already has D close to the plateau. On three networks (`Football`, `cintestinalis`, `bible_nouns`) the $M/10$ initialisation has not caught up to the σ_{lc} chain within the 5×10^4-step budget. In every case the chain initialised at σ_{lc} matches or exceeds the deeper-init chains, so using σ_{lc} as the standard initialisation never penalises the final D at fixed chain length.

For a stronger test we ran a height-based deep initialisation on `foodweb_baywet`, a network with a small improvement under adaptive cut ($D_{\text{lc}} \approx D_{\text{ac}} \approx 0.30$). Starting the chain at a dendrogram cut at similarity height 0.4 ($k_{\text{start}} \approx 562$, almost $7\times$ deeper than $k_{\text{lc}} = 81$) and running for 10^6 steps at $T = 10^{-4}$, all three seeds climb back up to within 0.06 of D_{lc}, with one trial within 0.002 (Table S4). The sampler recovers the single-level-cut plateau on this low-improvement network from a deep initialisation. We still use σ_{lc} in the main results because it converges in a small fraction of the steps and is a well-defined starting point.

Tables S3 and S4 report the numerical endpoints behind Fig. S3 and the height-based stress test.

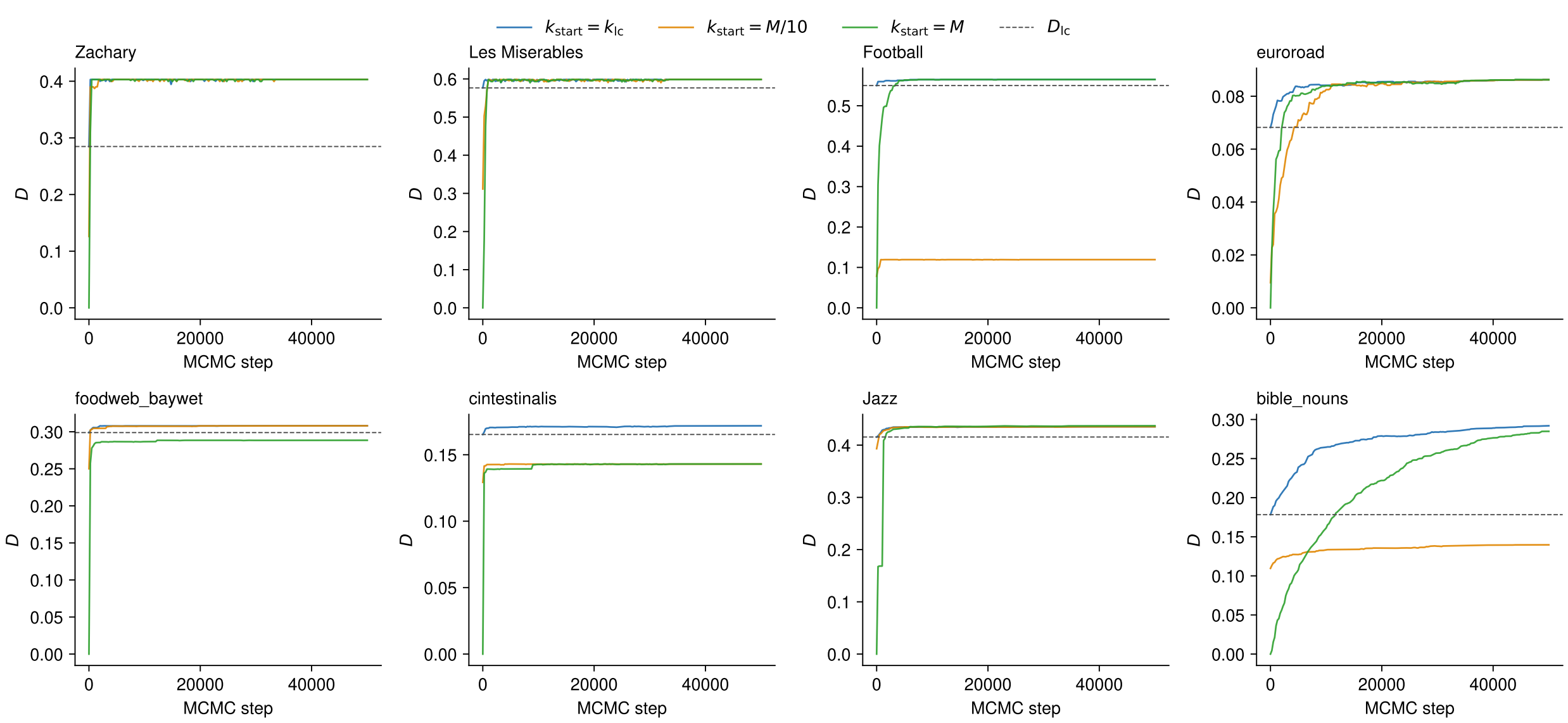

Figure S3: Effect of initialisation on D trajectories. Eight networks (same set as Fig. S4), three initialisations $k_{\text{start}} \in \{k_{\text{lc}}, M/10, M\}$, 5×10^4 steps, heuristic T_0. Dashed line: D_{lc}.

S5.3 Temperature sensitivity

We use a size- and balancedness-adaptive heuristic for the initial temperature,

$$T_0 = \frac{c}{M} f_B f_D, \quad f_B = \begin{cases} 3 & B < 0.15 \\ 1 & \text{otherwise} \end{cases}, \quad f_D = \begin{cases} 0.3 & D_{\text{lc}} > 0.7 \\ 0.01 & D_{\text{lc}} < 0.01 \\ 1 & \text{otherwise} \end{cases}, \tag{S5.1}$$

with $c = 0.1$ and the result clamped to $[10^{-8}, 10^{-3}]$. The factor $1/M$ keeps the acceptance rate roughly constant as network size grows; f_B raises T_0 on very unbalanced dendrograms where the chain must escape narrow basins; f_D lowers T_0 on near-saturated or near-zero landscapes where finer probes are needed.

We validated the heuristic in two ways. First, we swept T_0 over five orders of magnitude, $T_0 \in \{10^{-6}, 10^{-5}, 10^{-4}, 10^{-3}, 10^{-2}\}$, on eight representative networks at 2×10^5 steps with three trials each (Fig. S4). We also ran a $T_0 = 10^{-7}$ point on each network; the chain matches the 10^{-6} values to within noise and the corresponding curves are flat to the left of 10^{-6}, so we omit 10^{-7} from the plot. For every network, the heuristic temperature lies on the plateau of the $D(T_0)$ curve. Hotter values of T_0 do not help: at $T_0 = 10^{-2}$ most networks lose ground and D_{mcmc} falls below D_{lc} (negative improvement of 50–85% on the softer landscapes such as `foodweb_baywet`, `cintestinalis`, `Jazz`, and `bible_nouns`). Only the most "soft" landscapes benefit measurably from a colder T_0, and even then by less than 1%.

Second, we ran a $\{1\times, 10\times\}$ comparison around the heuristic T_0 on the same 25 networks used for convergence (Section S5.1). At 5×10^5 steps, the $1\times$ chain matched or beat the $10\times$ chain on all 25 networks (median gap 0.006, max gap 0.37): the chain at the heuristic T_0 has already settled while the hotter chain is still wandering. The heuristic is conservative: it is on the right side of the $D(T_0)$ plateau for all networks tested, and a $10\times$ hotter T_0 never improves the final D within 5×10^5 steps.

Table S5 reports the heuristic T_0 produced by Eq. S5.1 on the eight networks of Fig. S4.

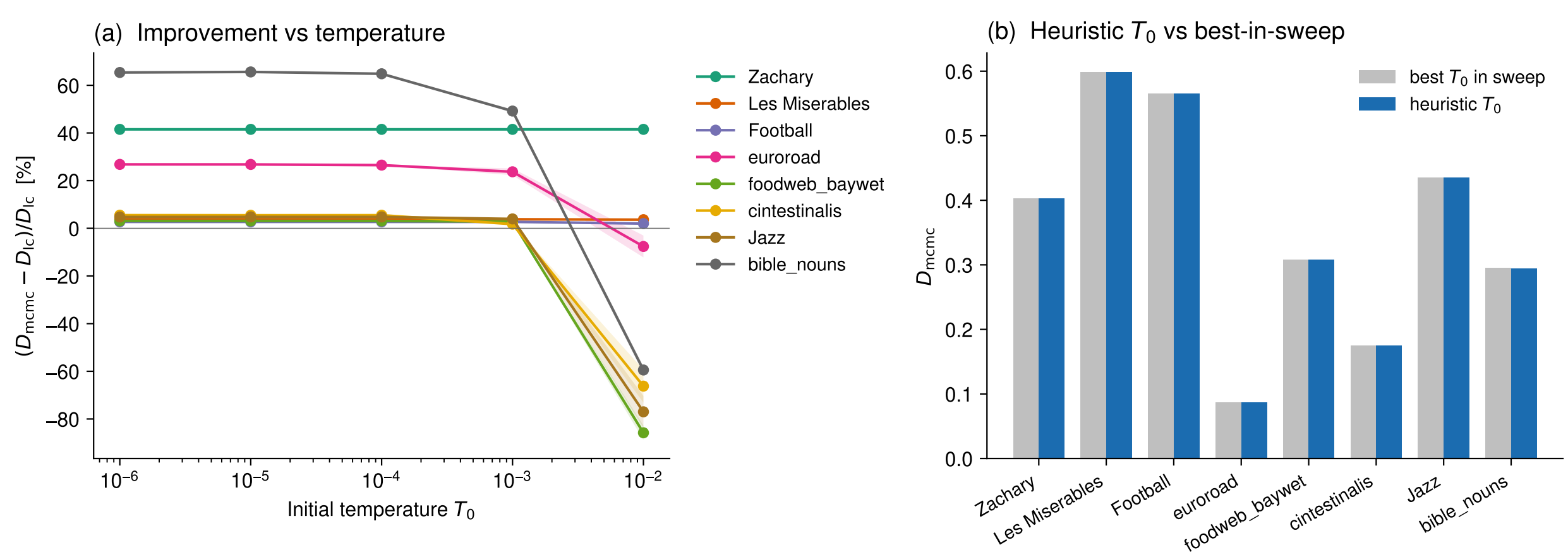

Figure S4: Temperature sensitivity. (a) Fractional improvement $D_{\text{mcmc}}/D_{\text{lc}}$ as a function of initial temperature T_0 for eight networks at 2×10^5 steps, mean over three trials. (b) For every network, the heuristic T_0 (Eq. S5.1, dashed) lies on the plateau of $D(T_0)$, so the heuristic matches the best T_0 in the sweep.

S5.4 Complexity and wall-clock timing

Before the MCMC begins, the sampler performs a one-off precomputation in $O(M)$ time: the geometric-cut normalisers $\log Z(v)$ and $\log Z_c(v)$, the leaf sets leaves(v), the down-move probability tables p_{stop} / p_{both} / $p_{\text{split},l/r}$, and the per-subtree partition-density contribution $D(v) = m_v \cdot D_c(m_v, n_v)$.

The per-step cost of the main loop is:

- UP-move: $O(k)$, where $k = |\sigma|$ is the current partition size. The dominant cost is the multi-collapse check, which scans current communities and tests disjointness against the sibling's leaf set; each test is amortised $O(\min(|\sigma_i|, |\text{leaves(sibling)}|))$.
- DOWN-move: $O(|\sigma'|)$, where $|\sigma'|$ is the size of the sampled cut. Sampling traverses the subtree top-down with constant-time tests against the precomputed probability tables.
- Acceptance / update: $O(|\sigma' - \sigma|)$ on accept, $O(1)$ on reject.

Because every partition community is by construction leaves(v) for some node v, the partition density is updated incrementally from the cached $D(v)$ values rather than recomputed from the raw edges, which keeps the per-step cost independent of the number of edges.

Table S6 reports the wall-clock breakdown on ten reference networks spanning two orders of magnitude in M, using a fixed seed, 2×10^5 MCMC steps, $T = 10^{-4}$, and a single CPU process (no parallelism). For every network in the table, total wall-clock is under 4 minutes, and dendrogram construction dominates the larger networks. `us_congress-H93` is the largest network in the table and finishes the MCMC in 18 s because its single-cut partition is already a strong local optimum (the chain stays close to σ_{lc}). Across the 202 networks, the wall-clock falls in the brackets: 1–10 s for $M < 1{,}000$, tens of seconds to a few minutes for $1{,}000 \leq M < 10{,}000$, and 1–10 min for $M \geq 10{,}000$ (a handful of large, high-improvement networks dominate the upper end).

S6 Balancedness as a Structural Descriptor

The balancedness B defined in the main text is correlated with adaptive-cut improvement (Fig. 4). This section reports two uses of B: (i) B as a practical decision criterion for whether to run adaptive cut on a given network, and (ii) B as a structural descriptor that characterises the network through the heterogeneity of its community structure. The manuscript's own VD-SBM example (Fig. 2) illustrates the second reading: the plain SBM with uniform community density gives $B = 0.23$, the varying-density SBM with the same number of communities but a density gradient across them gives $B = 0.03$. B tracks the heterogeneity of community sizes and densities, not just the number of communities.

S6.1 Decision criterion

We binned the 202 catalogue networks by balancedness and reported, per bin, the mean and median improvement over the single-level cut, and the fraction of networks with improvement of at least 5%, 10%, and 25% (Table S7 and Fig. S5). The mean improvement falls from 52.6% in the lowest bin ($B < 0.10$) to 16.4% at $B \in [0.25, 0.30)$. The fraction of networks with at least 25% improvement is 0.61 at $B < 0.10$ and drops to 0.12 at $B \in [0.25, 0.30)$. Networks with $B < 0.15$ have a $\geq 75\%$ chance of a $\geq 10\%$ improvement under adaptive cut.

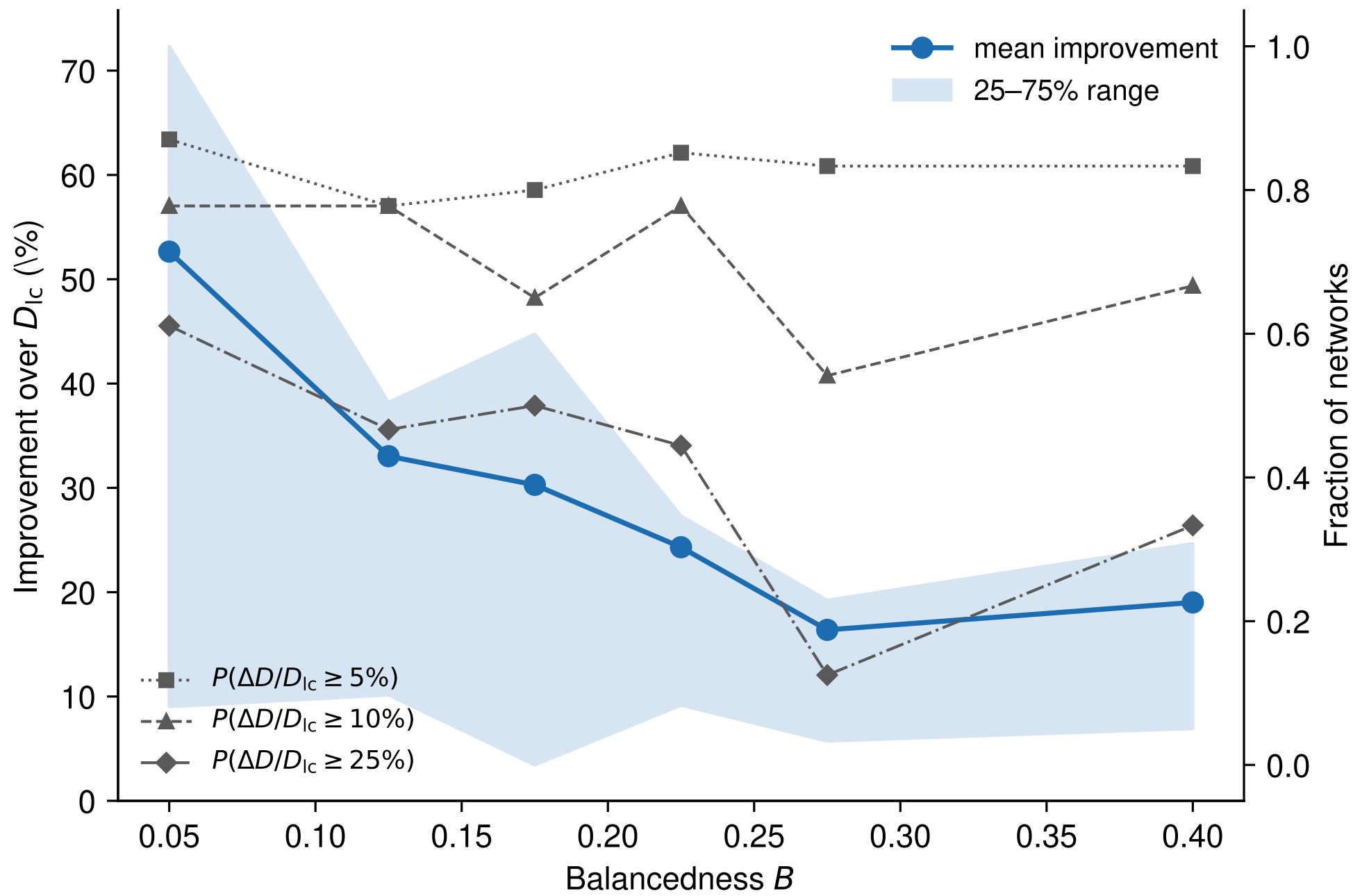

Figure S5: Decision criterion. Adaptive-cut improvement $(D_{\rm ac} - D_{\rm lc})/D_{\rm lc}$ vs B, binned: solid line is the mean per bin; band is the 25–75% range across networks. Dashed step lines (right axis) give the fraction of networks with improvement $\geq 5/10/25\%$.

S6.2 Partition disagreement, tree balance, and multiscale span

Beyond the predictor role, B relates to three structural axes of the dendrogram (Fig. S6; Table S8). First, the partition disagreement $1 - \mathrm{NMI}(\sigma_{\rm lc}, \sigma_{\rm ac})$ between the single-level cut and the adaptive cut falls as B rises (Pearson $r = -0.53$). Networks with low B admit single-level and multi-level partitions that disagree, supporting the use of B as a diagnostic for clustering reliability: when B is low, the single-level cut is not a stable representation of the dendrogram's community structure.

Second, B shows a weak positive correlation with the classical Colless ($r = +0.31$) and Sackin ($r = +0.37$) indices of leaf-balance. The Colless and Sackin indices are purely topological measures of how subtree sizes split at each internal node, while B aggregates entropy across levels of the dendrogram defined by similarity height. The weak correlation indicates that B captures dendrogram structure that classical leaf-balance indices do not fully describe; classical indices remain low on most link-clustering dendrograms (median $\mathrm{Colless}_{\rm norm} \approx 0.12$) while B spans the $[0, 0.36]$ range observed across the catalogue, revealing finer level-wise structure.

Third, the log span of community sizes in $\sigma_{\rm lc}$, $\log_{10}(\max m_c / \min m_c)$, decreases with B ($r = -0.28$). Low B coincides with $\sigma_{\rm lc}$ communities that span several orders of magnitude in edge count: the dendrogram contains communities at very different scales that a single horizontal cut cannot simultaneously resolve. This is the dendrogram-level analogue of the resolution-limit problem of single-resolution objectives [5, 6]. Standard tools for multiscale community detection, such as multiresolution modularity [7] and Markov-stability methods [8], address the same problem by sweeping a continuous resolution parameter or Markov time. Balancedness B describes the same multiscale property from the geometry of the link-clustering dendrogram, without a resolution sweep, and applies to any hierarchical clustering method that produces a dendrogram.

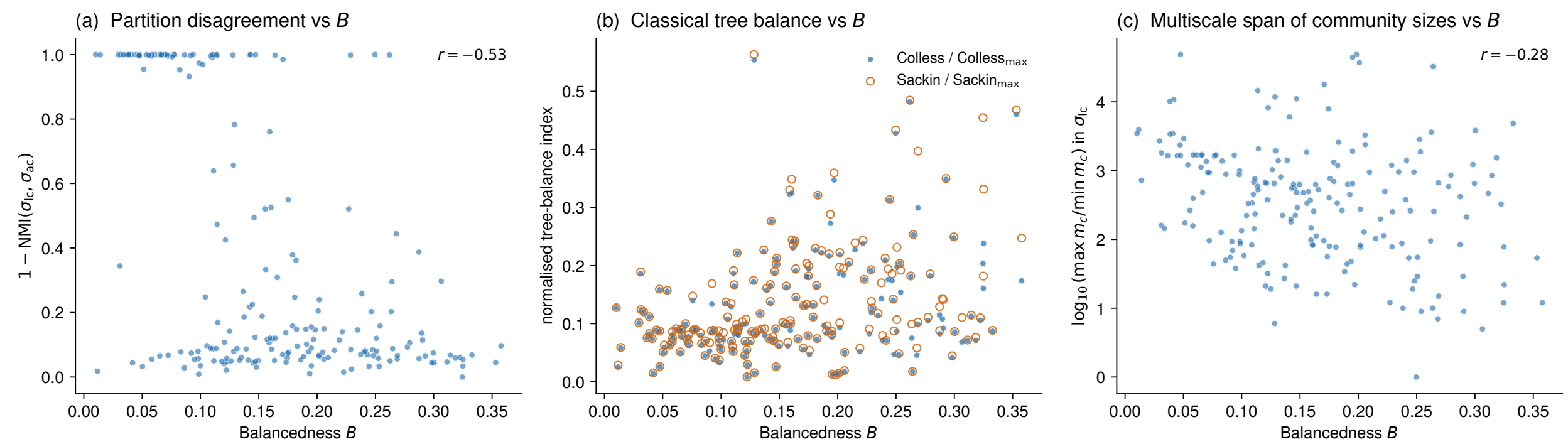

Figure S6: Balancedness as a structural descriptor. (a) Partition disagreement $1-\mathrm{NMI}(\sigma_{\mathrm{lc}}, \sigma_{\mathrm{ac}})$ vs B; low B implies single-level and multi-level partitions disagree. (b) Classical leaf-balance indices Colless and Sackin (normalised by their caterpillar maxima) vs B; the weak positive correlation shows that B captures dendrogram structure not fully described by topological leaf-balance. (c) $\log_{10}(\max m_c/\min m_c)$ over σ_{lc} communities vs B; low B networks carry communities spanning several orders of magnitude in edge count.

S6.3 What B captures: a controlled 2×2 design

The variable B is constructed from the level-wise partition entropy of the edge-similarity dendrogram. To identify what property of the network drives B, we crossed two binary axes on a single synthetic family: uniform vs varying intra-community density, and flat vs nested community structure. Each cell of the resulting 2×2 design is generated as a stochastic block model with 4 leaf communities of $N_c = 20$ nodes ($N = 80$ nodes total). Mean intra-density is fixed at $\bar{p}_{\mathrm{intra}} = 0.35$ in every cell. On the density axis, uniform cells use $p^{(c)}_{\mathrm{intra}} = 0.35$ for every community c; varying cells use $p^{(c)}_{\mathrm{intra}} \in \{0.65, 0.45, 0.25, 0.05\}$, the same mean and the same overall edge count M. On the nesting axis, flat cells use $p_{\mathrm{inter}} = 0.01$ between every pair of leaves; nested cells use $p_{\mathrm{inter}} = 0.10$ between leaves of the same super-community and $p_{\mathrm{inter}} = 0.005$ between leaves of different super-communities, where leaves $\{L0, L1\}$ form super-0 and $\{L2, L3\}$ form super-1.

For each cell we draw 5 independent networks, build the edge-similarity dendrogram with Jaccard similarity and single linkage, and compute B as in the main text. Table S9 reports the mean and standard deviation of B, the number of communities k_{lc} at the optimal single-level cut, and the network edge count M across the 5 seeds. Figure S7 shows, for seed 0 of each cell, the block-density matrix used to draw the SBM, the resulting dendrogram coloured by σ_{lc} with the optimal cut drawn as a dashed line, and the entropy curves $(H_{\min}, H_{\mathrm{real}}, H_{\max})$ at each similarity level.

The columns of the design (A vs B, C vs D) separate by 0.09–0.12 in B; the rows of the design (A vs C, B vs D) shift B by less than 0.04, within the seed-to-seed standard deviation ($n = 5$ seeds per cell). The density-variation axis is the dominant driver of B in this design; at this sample size we find no evidence for a nesting effect, but cannot rule out a small effect ($\lesssim 0.04$) that a larger seed budget might resolve. The entropy panels of Fig. S7 show the mechanism: in cells A and C the real entropy curve hugs $H_{\max}$ across most levels (so B is high); in cells B and D the real entropy is pulled down toward $H_{\min}$ across many levels (so B is low). Nesting changes the topology of the dendrogram (the nested cells C and D show two top-level super-branches, marked at the bottom of each dendrogram) but does not move the entropy curve appreciably.

Linkage sensitivity. The absolute value of B depends on the linkage method used to build the dendrogram. To test this we run a separate, larger version of the 2×2 design: eight leaves of $N_c = 25$ nodes (vs four leaves of $N_c = 20$ above) and a three-level nesting in the C and D cells (vs two-level above), with the same uniform/varying density crossing. On the same Jaccard edge-distance matrix per draw we build the dendrogram with single, average, and complete linkage. Across the four cells, single linkage on this larger graph chains the densest leaves together early and gives $B \in [0.12, 0.16]$ with no cell-to-cell signal above seed noise. Average and complete linkage give $B \in [0.57, 0.69]$ with the same cell ordering: density variation lowers B by about 0.05 under average linkage and about 0.02 under complete linkage, in both rows (Fig. S8, Table S10). The qualitative claim, low B tracks density variation, holds under average and complete linkage; under single linkage the chaining effect compresses B into the narrow band above and the cell signal is lost in seed noise, so both the absolute scale of B and the resolvability of the density signal are linkage-dependent. The values quoted in the main text (plain SBM $B = 0.23$, VD-SBM $B = 0.03$ in Fig. 2) are the single-linkage balancedness of those dendrograms; average and complete linkage would place the same block structures at a higher absolute scale, as this table shows.

Implications. Three points follow. (i) B measures dendrogram-level density heterogeneity, not nesting depth or hierarchical depth in any topological sense. (ii) B is related to multiscale community structure in only one direction: low B implies communities at different scales, so a single horizontal cut cannot resolve them all (this is the regime in which adaptive cut delivers most of its improvement). High B does not imply absence of multiscale structure. (iii) The connection to the resolution limit of [5] and to multi-resolution / Markov-stability methods [7, 8] is indirect: those methods target community size relative to total network scale through an objective-function or diffusion parameter, while B targets density heterogeneity from the geometry of a given dendrogram. The two are complementary diagnostics of the same multiscale phenomenon, computed from different objects.

S6.4 Relation to the Fernández–Gómez normalised tree balance

The closest published index to our balancedness is the Normalised Tree Balance (NTB) of Fernández & Gómez 2020 [9], defined (their Eqs. 18–20) on a dendrogram with M leaves as

$$H_I = -\sum_{i \in I} p_i \log_{|I|}(p_i), \quad p_i = \frac{|X_i|}{|X_I|}, \tag{S6.1}$$

$H = \text{mean}_I\, H_I$ averaged over the $M - 1$ internal nodes, and $\text{NTB} = (H - H_{\min})/(1 - H_{\min})$ where $H_{\min} = \frac{1}{M-1}\left[\log_2 M + \sum_{n=2}^{M-1} \frac{\log_2 n}{n+1}\right]$ is the value attained by the caterpillar binary tree of M leaves. For binary single-linkage dendrograms $|I| = 2$ everywhere, so each $H_I \in [0, 1]$ uses $\log_2$.

NTB and our B differ in how they aggregate balance: NTB averages a local Shannon entropy over internal *nodes* (a property of the topology), while B averages a normalised partition entropy over distinct similarity *levels* of the dendrogram. The latter is indexed along the dimension a horizontal cut runs over, and the weighted variant B_{weighted} further weighs each level by its similarity gap Δs.

We computed NTB on the 202 catalogue networks (`results/ntb_catalogue.csv`). The two indices are moderately correlated (Pearson $r = +0.45$, Spearman $\rho = +0.44$; Fig. S9a). The two indices capture related but distinct aspects of dendrogram balance. As predictors of adaptive-cut improvement (over D_{lc}) the two indices perform almost identically: Pearson(B, improv.) $= -0.38$ and Pearson(NTB, improv.) $= -0.40$ (Fig. S9b). NTB is marginally the stronger predictor on

this corpus, but the gap (0.02) is within the bootstrap noise. We keep B as the headline predictor because its level-wise indexing matches the object adaptive cut competes against: a single horizontal cut. NTB averages a local entropy over internal nodes regardless of similarity height, so it cannot distinguish a dendrogram whose communities sit at one similarity level from one whose communities are spread across many, and that spread is exactly what adaptive cut exploits.

Where the two indices diverge. Because NTB reads only the branching topology and B reads the similarity levels, the two can be driven apart by construction. Fix a perfectly balanced binary topology, for which NTB $= 1$ at every M, and vary only the heights at which its merges occur (Fig. S10a,b). When the merges form synchronised waves, every horizontal cut splits the leaves into equal blocks and $B \to 1$. When they form a staircase, with one subtree assembling before the next, every cut is dominated by a single growing block and B falls to 0.32 at $M = 128$, while NTB stays at 1 throughout. NTB cannot resolve the two cases because it never references merge heights, yet the difference is what decides whether a single horizontal cut suffices.

The opposite corner is unreachable. A caterpillar topology (NTB $\to 0$) forces every level partition to be one large cluster plus singletons, so B is small whenever NTB is small. Balanced topology is necessary but not sufficient for balanced levels, which confines the feasible (NTB, B) region to $B \lesssim$ NTB; on the 202 catalogue networks NTB $\geq B$ in 201 of 202 cases (the lone exception, an ego network, is a tie within 0.005; Fig. S9a). The networks farthest into the high-NTB/low-B corner are road networks, trees of life, and the yeast spliceosome (NTB $\approx$ 0.71–0.81, $B \approx$ 0.01–0.11): their single-linkage dendrograms split into comparable parts at most nodes (median child-size ratio $\approx$ 1:2, well away from the k:1 caterpillar) but collapse onto few distinct similarity levels, so no single cut resolves their communities (us_roads-VT: 20 distinct similarity levels across 10^5 merges, $B = 0.09$, NTB $= 0.81$). These are the dendrograms where adaptive cut gains the most, and the property that flags them, the spread of communities across cut levels, is the one B measures and NTB discards.

S7 Network Catalogue

Table S11 lists every network used to evaluate the adaptive cut in Figures 4 and 5 of the main text, with its domain, size (N, M), balancedness B, single-cut partition density D_{lc}, adaptive-cut partition density D_{ac}, relative improvement, source, and (where available) a clickable reference to the original publication.

Table S11: All 202 networks used to generate Figures 4 and 5, with their domain, size (N, M), balancedness B, single-cut D_{lc} and adaptive-cut D_{ac} partition densities, relative improvement, source, and original publication. Most networks come from the Netzschleuder catalog (`https://networks.skewed.de`); a smaller set from the graph-tool built-in collection (`https://graph-tool.skewed.de/static/doc/collection.html`); six classical benchmarks are cited individually. Improvement is $(D_{\text{ac}} - D_{\text{lc}})/D_{\text{lc}} \times 100\%$; entries shown as +0.0 improve by less than 0.05%. At full numerical precision 195 of the 202 networks strictly improve (7 are exact ties), and the mean (+34%) and median (+21%) improvements are computed at full precision.

#	Name	Domain	N	M	B	D_{lc}	D_{ac}	Impr.%	Source	Reference
1	Caenorhabditis elegans	biological	453	2,025	0.11	0.14	0.22	+50	graph-tool	Duch & Arenas (2005)
2	Clements & Long plant-pollinator web	biological	275	906	0.16	0.036	0.048	+32	graph-tool	Clements & Long (1923)
3	Messel Shale food web	biological	700	6,395	0.30	0.052	0.070	+35	graph-tool	Dunne et al. (2014)
4	Yeast spliceosome	biological	103	4,119	0.012	0.91	0.92	+0.0	graph-tool	Korkin et al. (2006)
5	budapest_connectome-all_200k	biological	1,015	104,292	0.20	0.39	0.39	+0.40	Netzschleuder	Szalkai et al. (2015)

continued on next page

Table S11 – continued from previous page

#	Name	Domain	N	M	B	$D_{\mathbf{lc}}$	$D_{\mathbf{ac}}$	Impr.%	Source	Reference
6	budapest_connectome-all_20k	biological	1,015	70,654	0.26	0.28	0.28	+0.27	Netzschleuder	Szalkai et al. (2015)
7	budapest_connectome-female_200k	biological	1,015	95,867	0.20	0.37	0.37	+0.17	Netzschleuder	Szalkai et al. (2015)
8	budapest_connectome-male_200k	biological	1,015	79,278	0.20	0.31	0.31	+0.16	Netzschleuder	Szalkai et al. (2015)
9	celegans_interactomes-wi2007	biological	1,108	1,500	0.11	0.013	0.021	+58	Netzschleuder	Simonis et al. (2009)
10	cintestinalis	biological	205	2,575	0.28	0.17	0.17	+5.5	Netzschleuder	Ryan (2016)
11	foodweb_baywet	biological	128	2,075	0.32	0.30	0.31	+3.1	Netzschleuder	Ulanowicz et al. (1998)
12	genetic_multiplex-Candida	biological	303	314	0.13	0.048	0.048	+0.0	Netzschleuder	De Domenico (2014)
13	genetic_multiplex-Gallus	biological	205	234	0.16	0.034	0.035	+2.9	Netzschleuder	De Domenico (2014)
14	genetic_multiplex-HumanHIV1	biological	1,005	1,155	0.12	0.00040	0.00040	+0.0	Netzschleuder	De Domenico (2014)
15	genetic_multiplex-Plasmodium	biological	1,158	2,402	0.16	0.0098	0.016	+66	Netzschleuder	De Domenico (2014)
16	genetic_multiplex-Rattus	biological	2,350	3,484	0.15	0.053	0.068	+30	Netzschleuder	De Domenico (2014)
17	human_brains-BNU1_0025864_1_DTI_AAL	biological	116	1,563	0.18	0.44	0.47	+6.8	Netzschleuder	Roncal et al. (2013)
18	human_brains-BNU1_0025864_1_DTI_CPAC200	biological	200	1,832	0.28	0.25	0.29	+18	Netzschleuder	Roncal et al. (2013)
19	human_brains-BNU1_0025864_1_DTI_DS00583	biological	540	7,425	0.33	0.21	0.23	+6.4	Netzschleuder	Roncal et al. (2013)
20	human_brains-BNU1_0025864_1_DTI_DS01216	biological	1,088	17,948	0.31	0.22	0.30	+33	Netzschleuder	Roncal et al. (2013)
21	human_brains-BNU1_0025870_1_DTI_DS01216	biological	1,133	16,478	0.30	0.24	0.29	+24	Netzschleuder	Roncal et al. (2013)
22	interactome_figeys	biological	2,217	6,418	0.086	0.0079	0.014	+75	Netzschleuder	Ewing et al. (2007)
23	interactome_pdz	biological	161	209	0.19	0.075	0.078	+3.6	Netzschleuder	Beuming et al. (2005)
24	interactome_stelzl	biological	1,615	3,106	0.14	0.010	0.015	+53	Netzschleuder	Stelzl et al. (2005)
25	interactome_vidal	biological	2,783	6,007	0.14	0.054	0.079	+46	Netzschleuder	JF. Rual et al. (2005)
26	interactome_yeast	biological	1,458	1,948	0.12	0.088	0.10	+15	Netzschleuder	Coulomb et al. (2005)
27	kegg_metabolic-aae	biological	880	2,296	0.18	0.045	0.076	+67	Netzschleuder	Huss (2007)
28	kegg_metabolic-afu	biological	861	2,011	0.17	0.036	0.052	+44	Netzschleuder	Huss (2007)
29	kegg_metabolic-ana	biological	1,314	3,552	0.15	0.030	0.051	+71	Netzschleuder	Huss (2007)
30	kegg_metabolic-ape	biological	769	1,858	0.20	0.030	0.057	+89	Netzschleuder	Huss (2007)
31	kegg_metabolic-atc	biological	1,538	4,315	0.16	0.028	0.052	+90	Netzschleuder	Huss (2007)
32	kegg_metabolic-ath	biological	1,531	4,285	0.13	0.027	0.039	+43	Netzschleuder	Huss (2007)
33	kegg_metabolic-atu	biological	1,542	4,323	0.18	0.028	0.051	+86	Netzschleuder	Huss (2007)
34	kegg_metabolic-bas	biological	619	1,501	0.22	0.052	0.075	+44	Netzschleuder	Huss (2007)
35	malaria_genes-HVR_1	biological	307	2,812	0.11	0.44	0.53	+20	Netzschleuder	Larremore et al. (2013)
36	malaria_genes-HVR_5	biological	298	2,684	0.19	0.26	0.35	+32	Netzschleuder	Larremore et al. (2013)
37	malaria_genes-HVR_6	biological	291	3,251	0.27	0.57	0.61	+7.0	Netzschleuder	Larremore et al. (2013)
38	malaria_genes-HVR_7	biological	306	11,688	0.21	0.59	0.64	+8.2	Netzschleuder	Larremore et al. (2013)
39	malaria_genes-HVR_8	biological	273	3,933	0.30	0.70	0.72	+3.0	Netzschleuder	Larremore et al. (2013)
40	malaria_genes-HVR_9	biological	297	7,562	0.25	0.50	0.53	+5.9	Netzschleuder	Larremore et al. (2013)
41	mist-genetic_fly	biological	4,055	13,779	0.27	0.046	0.073	+58	Netzschleuder	Yanhui Hu et al. (2017)
42	mist-genetic_human	biological	3,570	8,703	0.18	0.086	0.11	+23	Netzschleuder	Yanhui Hu et al. (2017)
43	mist-genetic_mouse	biological	214	252	0.29	0.12	0.12	+0.42	Netzschleuder	Yanhui Hu et al. (2017)
44	mist-genetic_worm	biological	3,037	7,914	0.18	0.056	0.071	+27	Netzschleuder	Yanhui Hu et al. (2017)
45	mist-ppi_rat	biological	6,803	14,636	0.17	0.023	0.037	+60	Netzschleuder	Yanhui Hu et al. (2017)
46	plant_pol_robertson	biological	1,882	15,254	0.23	0.017	0.023	+35	Netzschleuder	Robertson (1929)
47	tree-of-life-331678	biological	531	1,848	0.058	0.44	0.52	+18	Netzschleuder	Marinka Zitnik et al. (2019)
48	tree-of-life-333990	biological	178	282	0.25	0.26	0.29	+10	Netzschleuder	Marinka Zitnik et al. (2019)
49	tree-of-life-335543	biological	641	4,334	0.072	0.52	0.58	+13	Netzschleuder	Marinka Zitnik et al. (2019)
50	tree-of-life-338966	biological	595	3,140	0.098	0.48	0.55	+15	Netzschleuder	Marinka Zitnik et al. (2019)
51	tree-of-life-338969	biological	879	5,873	0.083	0.46	0.55	+20	Netzschleuder	Marinka Zitnik et al. (2019)
52	tree-of-life-339670	biological	1,360	9,646	0.12	0.45	0.53	+17	Netzschleuder	Marinka Zitnik et al. (2019)
53	tree-of-life-340177	biological	434	1,477	0.11	0.46	0.55	+20	Netzschleuder	Marinka Zitnik et al. (2019)

continued on next page

Table S11 – continued from previous page

#	Name	Domain	N	M	B	$D_{\mathbf{lc}}$	$D_{\mathbf{ac}}$	Impr.%	Source	Reference
54	tree-of-life-365044	biological	820	4,408	0.14	0.46	0.55	+18	Netzschleuder	Marinka Zitnik et al. (2019)
55	tree-of-life-366602	biological	861	4,970	0.15	0.44	0.51	+15	Netzschleuder	Marinka Zitnik et al. (2019)
56	tree-of-life-406817	biological	714	3,283	0.14	0.41	0.51	+23	Netzschleuder	Marinka Zitnik et al. (2019)
57	tree-of-life-452652	biological	1,021	7,516	0.12	0.47	0.54	+14	Netzschleuder	Marinka Zitnik et al. (2019)
58	tree-of-life-469383	biological	838	7,232	0.065	0.47	0.54	+17	Netzschleuder	Marinka Zitnik et al. (2019)
59	TrumpWorld	economic	2,669	3,380	0.031	0.024	0.029	+18	graph-tool	Buzzfeed News (2017)
60	bitcoin_alpha	economic	3,775	14,120	0.14	0.017	0.029	+76	Netzschleuder	Kumar et al. (2016)
61	bitcoin_trust	economic	5,875	21,489	0.16	0.023	0.039	+70	Netzschleuder	Kumar et al. (2016)
62	board_directors-net1m_2011-08-01	economic	854	2,745	0.099	0.85	0.92	+8.3	Netzschleuder	Seierstad et al. (2011)
63	board_directors-net2m_2011-08-01	economic	1,066	1,148	0.23	0.016	0.020	+26	Netzschleuder	Seierstad et al. (2011)
64	eu_procurements_alt-AT_2008	economic	1,684	1,921	0.099	0.0016	0.0040	+150	Netzschleuder	Wachs et al. (2020)
65	eu_procurements_alt-CZ_2008	economic	1,777	2,110	0.082	0.0017	0.0029	+73	Netzschleuder	Wachs et al. (2020)
66	eu_procurements_alt-DK_2011	economic	2,219	2,904	0.072	0.0028	0.0049	+75	Netzschleuder	Wachs et al. (2020)
67	eu_procurements_alt-EE_2008	economic	480	540	0.16	0.0065	0.0097	+49	Netzschleuder	Wachs et al. (2020)
68	eu_procurements_alt-ES_2008	economic	8,097	13,286	0.071	0.0019	0.0034	+79	Netzschleuder	Wachs et al. (2020)
69	eu_procurements_alt-FI_2008	economic	2,517	3,701	0.11	0.0047	0.0081	+72	Netzschleuder	Wachs et al. (2020)
70	eu_procurements_alt-GR_2008	economic	1,933	2,495	0.11	0.0015	0.0026	+73	Netzschleuder	Wachs et al. (2020)
71	eu_procurements_alt-HU_2015	economic	2,163	2,929	0.092	0.0062	0.010	+66	Netzschleuder	Wachs et al. (2020)
72	eu_procurements_alt-IT_2008	economic	6,622	9,121	0.11	0.0022	0.0040	+84	Netzschleuder	Wachs et al. (2020)
73	eu_procurements_alt-LT_2008	economic	1,359	2,486	0.14	0.015	0.020	+28	Netzschleuder	Wachs et al. (2020)
74	eu_procurements_alt-LV_2008	economic	1,628	2,269	0.051	0.0060	0.011	+75	Netzschleuder	Wachs et al. (2020)
75	eu_procurements_alt-PL_2008	economic	12,456	23,991	0.14	0.015	0.019	+23	Netzschleuder	Wachs et al. (2020)
76	eu_procurements_alt-PT_2008	economic	692	776	0.090	0.0038	0.0057	+51	Netzschleuder	Wachs et al. (2020)
77	eu_procurements_alt-SE_2008	economic	4,421	5,905	0.058	0.0024	0.0045	+88	Netzschleuder	Wachs et al. (2020)
78	eu_procurements_alt-SK_2008	economic	660	773	0.10	0.0090	0.011	+23	Netzschleuder	Wachs et al. (2020)
79	product_space-HS	economic	866	2,532	0.19	0.23	0.27	+18	Netzschleuder	Hausmann et al. (2014)
80	product_space-SITC	economic	774	1,779	0.16	0.22	0.26	+19	Netzschleuder	Hausmann et al. (2014)
81	us_agencies-alabama	economic	1,115	4,983	0.17	0.12	0.15	+22	Netzschleuder	Kosack et al. (2018)
82	us_agencies-florida	economic	2,010	27,570	0.17	0.34	0.36	+7.0	Netzschleuder	Kosack et al. (2018)
83	us_agencies-indiana	economic	1,017	6,505	0.25	0.15	0.17	+13	Netzschleuder	Kosack et al. (2018)
84	Oregon_0	informational	10,670	22,002	0.045	0.0063	0.011	+70	graph-tool	Leskovec et al. (2005, Oregon AS)
85	Oregon_1	informational	10,729	21,999	0.077	0.0060	0.010	+67	graph-tool	Leskovec et al. (2005, Oregon AS)
86	Oregon_2	informational	10,790	22,469	0.047	0.0071	0.011	+56	graph-tool	Leskovec et al. (2005, Oregon AS)
87	Oregon_3	informational	10,859	22,747	0.094	0.0070	0.011	+59	graph-tool	Leskovec et al. (2005, Oregon AS)
88	Oregon_4	informational	10,886	22,493	0.066	0.0060	0.011	+83	graph-tool	Leskovec et al. (2005, Oregon AS)
89	Oregon_5	informational	10,943	22,607	0.078	0.0065	0.010	+57	graph-tool	Leskovec et al. (2005, Oregon AS)
90	Oregon_6	informational	11,011	22,677	0.061	0.0060	0.010	+72	graph-tool	Leskovec et al. (2005, Oregon AS)
91	Oregon_7	informational	11,051	22,724	0.065	0.0059	0.010	+74	graph-tool	Leskovec et al. (2005, Oregon AS)
92	Political books	informational	105	441	0.33	0.29	0.31	+7.1	V. Krebs	Krebs (n.d.)
93	Route views	informational	6,474	12,572	0.070	0.0084	0.014	+69	graph-tool	University of Oregon Route Views (n.d.)
94	adjnoun	informational	112	425	0.15	0.063	0.096	+52	Netzschleuder	M.E.J. Newman (2006)
95	bible_nouns	informational	1,707	9,059	0.15	0.18	0.30	+66	Netzschleuder	Römhild
96	caida_as-20040105	informational	16,301	32,955	0.041	0.0068	0.011	+68	Netzschleuder	The CAIDA AS Relationships Dataset
97	caida_as-20050502	informational	19,489	39,859	0.059	0.0059	0.011	+80	Netzschleuder	The CAIDA AS Relationships Dataset
98	caida_as-20060116	informational	21,232	42,872	0.037	0.0060	0.010	+72	Netzschleuder	The CAIDA AS Relationships Dataset
99	caida_as-20070917	informational	8,020	18,203	0.030	0.0095	0.014	+46	Netzschleuder	The CAIDA AS Relationships Dataset
100	edit_wikibooks-aa	informational	23	23	0.33	0.0095	0.0095	+0.0	Netzschleuder	Wikimedia Foundation. Wikimedia downloads
101	edit_wikibooks-ar	informational	31,797	43,896	0.010	0.00060	0.0011	+83	Netzschleuder	Wikimedia Foundation. Wikimedia downloads
102	edit_wikibooks-ca	informational	7,767	15,421	0.11	0.0058	0.011	+98	Netzschleuder	Wikimedia Foundation. Wikimedia downloads
103	edit_wikibooks-id	informational	13,150	21,547	0.039	0.0016	0.0029	+81	Netzschleuder	Wikimedia Foundation. Wikimedia downloads
104	edit_wikibooks-sq	informational	6,534	7,491	0.047	0.00090	0.0016	+78	Netzschleuder	Wikimedia Foundation. Wikimedia downloads
105	edit_wikibooks-tr	informational	6,802	12,212	0.014	0.0037	0.0055	+49	Netzschleuder	Wikimedia Foundation. Wikimedia downloads
106	edit_wikibooks-uk	informational	2,833	4,020	0.066	0.0074	0.010	+40	Netzschleuder	Wikimedia Foundation. Wikimedia downloads
107	gnutella-04	informational	10,876	39,994	0.18	0.020	0.035	+79	Netzschleuder	Ripeanu et al. (2002)

continued on next page

Table S11 – continued from previous page

#	Name	Domain	N	M	B	$D_{\mathbf{lc}}$	$D_{\mathbf{ac}}$	Impr.%	Source	Reference
108	unicodelang	informational	858	1,249	0.076	0.016	0.024	+49	Netzschleuder	Kunegis (2016)
109	webkb-webkb_cornell_cocite	informational	346	13,416	0.12	0.76	0.79	+4.9	Netzschleuder	Slattery (1998)
110	webkb-webkb_texas_cocite	informational	334	16,494	0.042	0.78	0.79	+1.1	Netzschleuder	Slattery (1998)
111	webkb-webkb_washington_cocite	informational	434	15,231	0.15	0.72	0.73	+1.6	Netzschleuder	Slattery (1998)
112	webkb-webkb_wisconsin_cocite	informational	348	16,625	0.13	0.81	0.83	+2.2	Netzschleuder	Slattery (1998)
113	wiki_science	informational	677	6,517	0.31	0.36	0.39	+11	Netzschleuder	Calderone (2020)
114	word_adjacency-darwin	informational	7,377	44,205	0.11	0.0060	0.0086	+43	Netzschleuder	Milo. et al. (2004)
115	word_adjacency-french	informational	8,308	23,832	0.031	0.0010	0.0026	+160	Netzschleuder	Milo. et al. (2004)
116	word_adjacency-japanese	informational	2,698	7,995	0.054	0.0022	0.0039	+78	Netzschleuder	Milo. et al. (2004)
117	word_adjacency-spanish	informational	11,558	43,050	0.038	0.0020	0.0043	+115	Netzschleuder	Milo. et al. (2004)
118	CS-Aarhus_multiplex	social	61	353	0.24	0.42	0.44	+4.6	graph-tool	Magnani et al. (2013)
119	Ego facebook	social	2,888	2,981	0.11	0.00020	0.00020	+0.0	graph-tool	McAuley & Leskovec (2012)
120	Email	social	1,133	5,451	0.29	0.14	0.16	+17	graph-tool	Guimerà et al. (2003)
121	Epinions	social	964	14,214	0.26	0.33	0.40	+20	graph-tool	Richardson et al. (2003)
122	Erdos	social	6,927	11,850	0.056	0.0056	0.013	+139	graph-tool	Batagelj & Mrvar (2000)
123	Football	social	115	613	0.19	0.55	0.56	+2.7	Girvan & Newman (2002)	Girvan & Newman (2002)
124	Gallery Proximity	social	410	2,765	0.32	0.36	0.42	+15	graph-tool	Isella et al. (2011)
125	Haggle human proximity network	social	274	2,124	0.13	0.53	0.54	+2.3	graph-tool	Chaintreau et al. (2007)
126	High school dynamic contacts 1	social	126	1,709	0.16	0.45	0.48	+6.8	graph-tool	Mastrandrea et al. (2015)
127	High school dynamic contacts 2	social	180	2,220	0.19	0.33	0.33	+2.7	graph-tool	Fournet & Barrat (2014)
128	Hospital ward dynamic contacts	social	75	1,139	0.098	0.47	0.47	+1.2	graph-tool	Vanhems et al. (2013)
129	Hypertext 2009 dynamic contact network	social	113	2,196	0.16	0.37	0.37	+0.41	graph-tool	Isella et al. (2011)
130	Jazz	social	198	2,742	0.20	0.42	0.44	+5.2	graph-tool	Gleiser & Danon (2003)
131	Les Miserables	social	77	254	0.092	0.58	0.60	+3.8	Knuth (1993)	Knuth (1993)
132	Pretty Good Privacy	social	10,680	24,316	0.13	0.26	0.36	+35	graph-tool	Bogu\{}˜{n}\{}'{a} et al. (2004)
133	STEM concept networks Researchers	social	1,616	3,045	0.14	0.019	0.045	+132	graph-tool	Koponen & Nousiainen (2014)
134	Song of ice and fire	social	796	2,823	0.16	0.12	0.18	+49	graph-tool	Beveridge & Shan (2016)
135	Star Wars	social	109	398	0.17	0.32	0.38	+20	Gabasova (2016)	Gabasova (2016)
136	Workplace contacts	social	92	755	0.20	0.33	0.33	+2.4	graph-tool	Génois et al. (2015)
137	Zachary	social	34	78	0.33	0.28	0.40	+42	Zachary (1977)	Zachary (1977)
138	arxiv_authors-GrQc	social	4,158	13,422	0.13	0.49	0.60	+23	Netzschleuder	Leskovec et al. (2007)
139	arxiv_authors-HepTh	social	8,638	24,806	0.22	0.29	0.37	+29	Netzschleuder	Leskovec et al. (2007)
140	arxiv_collab-hep-th-1999	social	5,835	13,815	0.23	0.35	0.43	+24	Netzschleuder	Newman (2001)
141	copenhagen-fb_friends	social	800	6,418	0.24	0.15	0.21	+44	Netzschleuder	Sapiezynski et al. (2019)
142	ego_social-facebook_0	social	324	2,514	0.23	0.32	0.35	+10	Netzschleuder	McAuley & Leskovec (2014)
143	ego_social-facebook_348	social	224	3,192	0.20	0.36	0.37	+3.3	Netzschleuder	McAuley & Leskovec (2012)
144	facebook_friends	social	329	1,954	0.29	0.41	0.44	+5.7	Netzschleuder	Maier et al. (2017)
145	facebook_organizations-L1	social	5,793	30,753	0.24	0.13	0.15	+15	Netzschleuder	Fire et al. (2016)
146	facebook_organizations-M1	social	1,429	19,357	0.25	0.13	0.15	+18	Netzschleuder	Fire et al. (2016)
147	facebook_organizations-S1	social	320	2,369	0.24	0.17	0.20	+13	Netzschleuder	Fire et al. (2016)
148	facebook_organizations-S2	social	165	726	0.21	0.22	0.26	+17	Netzschleuder	Fire et al. (2016)
149	foursquare-NYC_restaurant_checkin	social	4,906	13,457	0.15	0.0025	0.0047	+90	Netzschleuder	Yang et al. (2013)
150	foursquare-NYC_restaurant_tips	social	5,372	8,852	0.034	0.00090	0.0028	+211	Netzschleuder	Yang et al. (2013)
151	marvel_universe	social	19,182	95,445	0.21	0.040	0.059	+46	Netzschleuder	Alberich et al. (2002)
152	new_zealand_collab	social	1,463	4,246	0.10	0.060	0.083	+37	Netzschleuder	Aref et al. (2018)
153	physics_collab-pierreAuger	social	475	6,426	0.050	0.88	0.90	+2.8	Netzschleuder	De Domenico et al. (2015)
154	student_cooperation	social	141	256	0.26	0.76	0.77	+1.4	Netzschleuder	Fire et al. (2012)
155	ugandan_village-friendship-1	social	202	547	0.20	0.071	0.10	+46	Netzschleuder	Chami et al. (2017)
156	ugandan_village-friendship-2	social	181	688	0.35	0.081	0.13	+63	Netzschleuder	Chami et al. (2017)

continued on next page

Table S11 – continued from previous page

#	Name	Domain	N	M	B	$D_{\mathbf{lc}}$	$D_{\mathbf{ac}}$	Impr.%	Source	Reference
157	ugandan_village-friendship-3	social	192	1,060	0.25	0.048	0.082	+71	Netzschleuder	Chami et al. (2017)
158	ugandan_village-friendship-4	social	320	2,076	0.26	0.035	0.063	+79	Netzschleuder	Chami et al. (2017)
159	us_congress-H100	social	446	50,688	0.048	0.51	0.51	+0.0	Netzschleuder	Neal (1973)
160	us_congress-H93	social	446	18,083	0.17	0.18	0.18	+0.0	Netzschleuder	Neal (1973)
161	us_congress-S100	social	101	2,143	0.11	0.42	0.42	+0.0	Netzschleuder	Neal (1973)
162	us_congress-S97	social	101	2,073	0.13	0.41	0.41	+0.0	Netzschleuder	Neal (1973)
163	Power grid	technological	4,941	6,594	0.17	0.14	0.18	+29	graph-tool	Watts & Strogatz (1998)
164	internet_top_pop-Cogentco	technological	197	243	0.31	0.065	0.073	+14	Netzschleuder	Knight et al. (2011)
165	internet_top_pop-Kdl	technological	754	895	0.27	0.071	0.076	+6.4	Netzschleuder	Knight et al. (2011)
166	internet_top_pop-Ntt	technological	32	63	0.27	0.24	0.28	+14	Netzschleuder	Knight et al. (2011)
167	internet_top_pop-TataNld	technological	145	186	0.29	0.15	0.17	+14	Netzschleuder	Knight et al. (2011)
168	software_dependencies-colt	technological	504	1,139	0.20	0.14	0.17	+25	Netzschleuder	Šubelj (2012)
169	software_dependencies-jmail	technological	192	488	0.24	0.22	0.26	+18	Netzschleuder	Šubelj (2012)
170	software_dependencies-jung	technological	398	943	0.12	0.16	0.21	+30	Netzschleuder	Šubelj (2012)
171	software_dependencies-jung-c	technological	879	2,047	0.22	0.14	0.18	+31	Netzschleuder	Šubelj (2012)
172	software_dependencies-org	technological	486	2,717	0.11	0.13	0.18	+42	Netzschleuder	Šubelj (2012)
173	software_dependencies-sjbullet	technological	249	802	0.20	0.18	0.21	+15	Netzschleuder	Šubelj (2012)
174	software_dependencies-sjung	technological	399	946	0.14	0.16	0.21	+33	Netzschleuder	Šubelj (2012)
175	software_dependencies-slucene	technological	2,811	10,741	0.18	0.092	0.13	+40	Netzschleuder	Šubelj (2012)
176	Central Chilean power grid_reduced	transportation	218	527	0.23	0.54	0.61	+12	graph-tool	Kim et al. (2018)
177	Chicago road network	transportation	12,979	20,627	0.16	0.12	0.17	+37	graph-tool	Eash et al. (1979)
178	EUAirTransportation_multiplex	transportation	417	2,953	0.21	0.17	0.19	+8.0	graph-tool	Cardillo et al. (2013)
179	Train	transportation	64	243	0.25	0.56	0.63	+13	graph-tool	De Domenico et al. (2014)
180	US airport network	transportation	500	2,980	0.14	0.24	0.27	+12	graph-tool	Colizza et al. (2006)
181	contiguous_usa	transportation	49	107	0.36	0.28	0.35	+25	Netzschleuder	Knuth (1993)
182	euroroad	transportation	1,039	1,305	0.24	0.068	0.086	+27	Netzschleuder	Šubelj (2011)
183	london_transport	transportation	369	430	0.26	0.092	0.11	+20	Netzschleuder	Manlio De Domenico et al. (2014)
184	openstreetmap-01-AL-counties-street_networks:01017_Chambers_County	transportation	3,947	4,935	0.12	0.13	0.15	+11	Netzschleuder	Boeing (2018)
185	openstreetmap-01-AL-counties-street_networks:01031_Coffee_County	transportation	3,844	5,079	0.12	0.16	0.18	+14	Netzschleuder	Boeing (2018)
186	openstreetmap-01-AL-counties-street_networks:01121_Talladega_County	transportation	9,877	11,843	0.095	0.11	0.13	+13	Netzschleuder	Boeing (2018)
187	urban_streets-ahmedabad	transportation	2,870	4,375	0.16	0.16	0.17	+9.1	Netzschleuder	Crucitti et al. (2006)
188	urban_streets-bologna	transportation	541	771	0.25	0.12	0.13	+4.5	Netzschleuder	Crucitti et al. (2006)
189	urban_streets-brasilia	transportation	179	230	0.25	0.11	0.11	+6.0	Netzschleuder	Crucitti et al. (2006)
190	urban_streets-cairo	transportation	1,496	2,252	0.17	0.15	0.16	+8.1	Netzschleuder	Crucitti et al. (2006)
191	urban_streets-paris	transportation	335	494	0.25	0.14	0.17	+24	Netzschleuder	Crucitti et al. (2006)
192	urban_streets-venice	transportation	1,840	2,397	0.19	0.11	0.13	+14	Netzschleuder	Crucitti et al. (2006)
193	urban_streets-vienna	transportation	467	691	0.29	0.087	0.092	+5.8	Netzschleuder	Crucitti et al. (2006)
194	us_roads-AK	transportation	48,560	55,014	0.12	0.046	0.053	+16	Netzschleuder	9th DIMACS Implementation Challenge - Shortest Paths
195	us_roads-CT	transportation	152,036	184,393	0.055	0.086	0.090	+5.4	Netzschleuder	9th DIMACS Implementation Challenge - Shortest Paths
196	us_roads-DC	transportation	9,522	14,807	0.15	0.13	0.17	+28	Netzschleuder	9th DIMACS Implementation Challenge - Shortest Paths
197	us_roads-DE	transportation	48,812	59,502	0.086	0.076	0.091	+19	Netzschleuder	9th DIMACS Implementation Challenge - Shortest Paths
198	us_roads-LA	transportation	408,161	489,985	0.046	0.058	0.058	+0.17	Netzschleuder	9th DIMACS Implementation Challenge - Shortest Paths
199	us_roads-MT	transportation	300,809	343,444	0.098	0.052	0.057	+9.4	Netzschleuder	9th DIMACS Implementation Challenge - Shortest Paths
200	us_roads-NH	transportation	115,055	130,197	0.066	0.063	0.069	+9.9	Netzschleuder	9th DIMACS Implementation Challenge - Shortest Paths
201	us_roads-RI	transportation	51,642	66,650	0.10	0.088	0.11	+19	Netzschleuder	9th DIMACS Implementation Challenge - Shortest Paths
202	us_roads-VT	transportation	95,672	104,644	0.094	0.049	0.053	+8.6	Netzschleuder	9th DIMACS Implementation Challenge - Shortest Paths

S8 Adaptive Cut on the Louvain Dendrogram

Table S12 lists, for each of the 202 networks, the modularity of the single-seed Louvain partition Q_{Louvain} and of the adaptive cut applied to the Louvain dendrogram Q_{AC}, both measured at resolution $\gamma = 1$, with the relative improvement plotted in Figure 5.

Table S12: Modularity of the single-seed Louvain partition Q_{Louvain} and of the adaptive cut applied to the Louvain dendrogram Q_{AC}, for the 202 networks of Figure 4, both at resolution $\gamma = 1$. Improvement is $(Q_{\text{AC}} - Q_{\text{Louvain}})/Q_{\text{Louvain}} \times 100\%$.

**Network**	Q_{Louvain}	Q_{AC}	**Impr.%**
adjnoun	0.3016	0.3016	+0.0
arxiv_authors-GrQc	0.8470	0.8510	+0.5
arxiv_authors-HepTh	0.7555	0.7555	+0.0
arxiv_collab-hep-th-1999	0.8249	0.8275	+0.3
bible_nouns	0.5065	0.5251	+3.7
bitcoin_alpha	0.4698	0.4799	+2.1
bitcoin_trust	0.4829	0.5065	+4.9
board_directors-net1m_2011-08-01	0.8795	0.8815	+0.2
board_directors-net2m_2011-08-01	0.9101	0.9101	+0.0
budapest_connectome-all_200k	0.4965	0.4965	+0.0
budapest_connectome-all_20k	0.5574	0.5574	+0.0
budapest_connectome-female_200k	0.5035	0.5035	+0.0
budapest_connectome-male_200k	0.5551	0.5551	+0.0
Caenorhabditis elegans	0.4370	0.4457	+2.0
caida_as-20040105	0.6512	0.6608	+1.5
caida_as-20050502	0.6582	0.6695	+1.7
caida_as-20060116	0.6554	0.6751	+3.0
caida_as-20070917	0.5479	0.5546	+1.2
Central Chilean power grid_reduced	0.7785	0.7789	+0.0
Chicago road network	0.9310	0.9334	+0.3
cintestinalis	0.3414	0.3419	+0.1
Clements & Long plant-pollinator web	0.3113	0.3113	+0.0
Contact	0.1274	0.1342	+5.3
contiguous_usa	0.5811	0.5844	+0.6
copenhagen-fb_friends	0.4684	0.4762	+1.7
CS-Aarhus_multiplex	0.4162	0.4162	+0.0
David Copperfield	0.3008	0.3024	+0.5
edit_wikibooks-ar	0.6200	0.6217	+0.3
edit_wikibooks-ca	0.6192	0.6326	+2.2
edit_wikibooks-id	0.6391	0.6465	+1.2
edit_wikibooks-sq	0.7419	0.7425	+0.1
edit_wikibooks-tr	0.6192	0.6348	+2.5
edit_wikibooks-uk	0.7370	0.7375	+0.1
Ego facebook	0.8087	0.8087	+0.0
Email	0.5715	0.5780	+1.1
Email Europe	0.4107	0.4175	+1.7
Epinions	0.5294	0.5358	+1.2
Erdos	0.6959	0.6970	+0.2
eu_procurements_alt-AT_2008	0.8622	0.8641	+0.2
eu_procurements_alt-CZ_2008	0.8407	0.8407	+0.0
eu_procurements_alt-DK_2011	0.7803	0.7849	+0.6

continued on next page

Table S12 – continued from previous page

Network	Q_{Louvain}	Q_{AC}	Impr.%
eu_procurements_alt-EE_2008	0.8266	0.8266	+0.0
eu_procurements_alt-ES_2008	0.7271	0.7280	+0.1
eu_procurements_alt-FI_2008	0.7194	0.7194	+0.0
eu_procurements_alt-GR_2008	0.8029	0.8033	+0.1
eu_procurements_alt-HU_2015	0.8029	0.8046	+0.2
eu_procurements_alt-IT_2008	0.7843	0.7920	+1.0
eu_procurements_alt-LT_2008	0.6607	0.6648	+0.6
eu_procurements_alt-LV_2008	0.7924	0.7942	+0.2
eu_procurements_alt-PL_2008	0.7202	0.7235	+0.5
eu_procurements_alt-PT_2008	0.8619	0.8619	+0.0
eu_procurements_alt-SE_2008	0.7961	0.7976	+0.2
eu_procurements_alt-SK_2008	0.8319	0.8359	+0.5
EUAirTransportation_multiplex	0.2964	0.3079	+3.9
euroroad	0.8661	0.8661	+0.0
facebook_friends	0.6998	0.7072	+1.1
facebook_organizations-L1	0.7576	0.7591	+0.2
facebook_organizations-M1	0.5098	0.5134	+0.7
facebook_organizations-S1	0.4467	0.4542	+1.7
facebook_organizations-S2	0.5108	0.5153	+0.9
foodweb_baywet	0.1793	0.1820	+1.5
Football	0.5978	0.6046	+1.1
foursquare-NYC_restaurant_checkin	0.4365	0.4365	+0.0
foursquare-NYC_restaurant_tips	0.6332	0.6356	+0.4
Gallery Proximity	0.7048	0.7121	+1.0
genetic_multiplex-Candida	0.4544	0.4544	+0.0
genetic_multiplex-Gallus	0.4900	0.4900	+0.0
genetic_multiplex-HumanHerpes4	0.3216	0.3242	+0.8
genetic_multiplex-HumanHIV1	0.7179	0.7179	+0.0
genetic_multiplex-Plasmodium	0.5122	0.5122	+0.0
genetic_multiplex-Rattus	0.7150	0.7194	+0.6
gnutella-04	0.3816	0.3840	+0.6
Haggle human proximity network	0.1274	0.1315	+3.2
High school dynamic contacts 1	0.4587	0.4587	+0.0
High school dynamic contacts 1 copy	0.4587	0.4587	+0.0
High school dynamic contacts 2	0.5232	0.5232	+0.0
Hospital ward dynamic contacts	0.0973	0.0981	+0.9
human_brains-BNU1_0025864_1_DTI_AAL	0.3766	0.3796	+0.8
human_brains-BNU1_0025864_1_DTI_CPAC200	0.4465	0.4518	+1.2
human_brains-BNU1_0025864_1_DTI_DS00583	0.4544	0.4611	+1.5
human_brains-BNU1_0025864_1_DTI_DS01216	0.5140	0.5179	+0.7
human_brains-BNU1_0025870_1_DTI_DS01216	0.5439	0.5572	+2.5
Hypertext 2009 dynamic contact network	0.1011	0.1061	+5.0
interactome_figeys	0.4581	0.4638	+1.2
interactome_stelzl	0.5873	0.5873	+0.0
interactome_vidal	0.6361	0.6361	+0.0
internet_top_pop-Cogentco	0.8047	0.8047	+0.0
internet_top_pop-Kdl	0.8812	0.8851	+0.4
internet_top_pop-Ntt	0.4519	0.4544	+0.5
internet_top_pop-TataNld	0.7599	0.7641	+0.6
Jazz	0.4449	0.4451	+0.1
kegg_metabolic-aae	0.5150	0.5227	+1.5
kegg_metabolic-afu	0.5479	0.5479	+0.0

continued on next page

Table S12 – continued from previous page

Network	$Q_{\textbf{Louvain}}$	$Q_{\textbf{AC}}$	**Impr.%**
kegg_metabolic-ana	0.5191	0.5191	+0.0
kegg_metabolic-ape	0.5344	0.5395	+1.0
kegg_metabolic-atc	0.5099	0.5099	+0.0
kegg_metabolic-ath	0.5114	0.5114	+0.0
kegg_metabolic-atu	0.5038	0.5144	+2.1
kegg_metabolic-bas	0.5527	0.5580	+1.0
Les Miserables	0.5600	0.5600	+0.0
london_transport	0.8297	0.8297	+0.0
malaria_genes-HVR_1	0.6366	0.6399	+0.5
malaria_genes-HVR_5	0.4175	0.4371	+4.7
malaria_genes-HVR_6	0.5189	0.5298	+2.1
malaria_genes-HVR_7	0.3748	0.3777	+0.8
malaria_genes-HVR_8	0.5031	0.5041	+0.2
malaria_genes-HVR_9	0.3662	0.3706	+1.2
marvel_universe	0.6877	0.6922	+0.7
Messel Shale food web	0.3242	0.3604	+11.2
mist-genetic_fly	0.5051	0.5225	+3.4
mist-genetic_human	0.7389	0.7397	+0.1
mist-genetic_mouse	0.8141	0.8143	+0.0
mist-genetic_worm	0.5846	0.6070	+3.8
mist-ppi_rat	0.6236	0.6353	+1.9
name	0.4684	0.4762	+1.7
new_zealand_collab	0.4619	0.4719	+2.2
openstreetmap-01-AL-counties-street_networks:01017_Chambers_County	0.9429	0.9429	+0.0
openstreetmap-01-AL-counties-street_networks:01031_Coffee_County	0.9391	0.9394	+0.0
openstreetmap-01-AL-counties-street_networks:01121_Talladega_County	0.9615	0.9615	+0.0
Oregon_0	0.6278	0.6395	+1.9
Oregon_1	0.6271	0.6392	+1.9
Oregon_2	0.6231	0.6340	+1.7
Oregon_3	0.6201	0.6330	+2.1
Oregon_4	0.6209	0.6329	+1.9
Oregon_5	0.6224	0.6343	+1.9
Oregon_6	0.6255	0.6357	+1.6
Oregon_7	0.6169	0.6348	+2.9
physics_collab-pierreAuger	0.6144	0.6147	+0.0
plant_pol_robertson	0.3542	0.3565	+0.6
Political books	0.5268	0.5272	+0.1
Power grid	0.9359	0.9382	+0.3
Pretty Good Privacy	0.8824	0.8846	+0.3
product_space-HS	0.7153	0.7170	+0.2
product_space-SITC	0.7529	0.7580	+0.7
Route views	0.6212	0.6353	+2.3
software_dependencies-colt	0.6399	0.6436	+0.6
software_dependencies-jmail	0.6053	0.6121	+1.1
software_dependencies-jung	0.7425	0.7436	+0.1
software_dependencies-jung-c	0.7625	0.7674	+0.6
software_dependencies-org	0.4279	0.4320	+1.0
software_dependencies-scolt	0.6400	0.6410	+0.2
software_dependencies-sjbullet	0.5725	0.5750	+0.4
software_dependencies-sjung	0.7373	0.7373	+0.0
software_dependencies-slucene	0.7127	0.7194	+0.9
Song of ice and fire	0.5899	0.6114	+3.6

continued on next page

Table S12 – continued from previous page

**Network**	$Q_{\mathbf{Louvain}}$	$Q_{\mathbf{AC}}$	**Impr.%**
Star Wars	0.4274	0.4274	+0.0
STEM concept networks Researchers	0.5847	0.5895	+0.8
student_cooperation	0.8064	0.8064	+0.0
Train	0.4382	0.4421	+0.9
tree-of-life-331678	0.7491	0.7508	+0.2
tree-of-life-333990	0.8244	0.8249	+0.1
tree-of-life-335543	0.5880	0.5890	+0.2
tree-of-life-338966	0.6405	0.6523	+1.8
tree-of-life-338969	0.6638	0.6673	+0.5
tree-of-life-339670	0.7326	0.7396	+0.9
tree-of-life-340177	0.7504	0.7504	-0.0
tree-of-life-365044	0.7607	0.7619	+0.2
tree-of-life-366602	0.7274	0.7319	+0.6
tree-of-life-406817	0.7327	0.7372	+0.6
tree-of-life-452652	0.7356	0.7381	+0.3
tree-of-life-469383	0.6577	0.6588	+0.2
TrumpWorld	0.8018	0.8027	+0.1
ugandan_village-friendship-1	0.4219	0.4219	+0.0
ugandan_village-friendship-2	0.3293	0.3293	+0.0
ugandan_village-friendship-3	0.2574	0.2574	+0.0
ugandan_village-friendship-4	0.2848	0.2936	+3.1
unicodelang	0.7191	0.7191	+0.0
urban_streets-ahmedabad	0.9074	0.9082	+0.1
urban_streets-bologna	0.8327	0.8327	+0.0
urban_streets-brasilia	0.7898	0.7898	+0.0
urban_streets-cairo	0.8859	0.8859	+0.0
urban_streets-paris	0.7983	0.7983	+0.0
urban_streets-venice	0.9189	0.9222	+0.4
urban_streets-vienna	0.8107	0.8126	+0.2
US airport network	0.3559	0.3663	+2.9
us_agencies-alabama	0.5059	0.5167	+2.1
us_agencies-florida	0.5413	0.5427	+0.3
us_agencies-indiana	0.4101	0.4150	+1.2
us_congress-H100	0.0921	0.0934	+1.4
us_congress-H93	0.1449	0.1489	+2.8
us_congress-S100	0.1160	0.1287	+11.0
us_congress-S97	0.1213	0.1233	+1.6
us_roads-AK	0.9856	0.9856	+0.0
us_roads-CT	0.9852	0.9852	+0.0
us_roads-DC	0.9402	0.9402	+0.0
us_roads-DE	0.9793	0.9793	+0.0
us_roads-LA	0.9917	0.9917	+0.0
us_roads-MT	0.9921	0.9921	+0.0
us_roads-NH	0.9864	0.9867	+0.0
us_roads-RI	0.9801	0.9805	+0.0
us_roads-VT	0.9851	0.9853	+0.0
webkb-webkb_cornell_cocite	0.2503	0.2569	+2.6
webkb-webkb_texas_cocite	0.2184	0.2188	+0.2
webkb-webkb_washington_cocite	0.2633	0.2659	+1.0
webkb-webkb_wisconsin_cocite	0.2239	0.2257	+0.8
wiki_science	0.6199	0.6213	+0.2
word_adjacency-darwin	0.2393	0.2393	+0.0

continued on next page

Table S12 – continued from previous page

Network	$Q_{\mathbf{Louvain}}$	$Q_{\mathbf{AC}}$	Impr.%
word_adjacency-french	0.4041	0.4082	+1.0
word_adjacency-japanese	0.3670	0.3674	+0.1
word_adjacency-spanish	0.3179	0.3457	+8.7
Workplace contacts	0.3857	0.3857	+0.0
Zachary	0.4198	0.4198	+0.0

References

[1] Peter J. Green. Reversible jump Markov chain Monte Carlo computation and Bayesian model determination. *Biometrika*, 82(4):711–732, 1995.

[2] David A. Levin, Yuval Peres, and Elizabeth L. Wilmer. *Markov Chains and Mixing Times*. American Mathematical Society, Providence, RI, 2nd edition, 2017.

[3] Bruce Hajek. Cooling schedules for optimal annealing. *Mathematics of Operations Research*, 13(2):311–329, 1988.

[4] Peter Langfelder, Bin Zhang, and Steve Horvath. Defining clusters from a hierarchical cluster tree: the dynamic tree cut package for r. *Bioinformatics*, 24(5):719–720, 2008.

[5] Santo Fortunato and Marc Barthelemy. Resolution limit in community detection. *Proceedings of the national academy of sciences*, 104(1):36–41, 2007.

[6] Andrea Lancichinetti and Santo Fortunato. Limits of modularity maximization in community detection. *Physical Review E—Statistical, Nonlinear, and Soft Matter Physics*, 84(6):066122, 2011.

[7] Jörg Reichardt and Stefan Bornholdt. Statistical mechanics of community detection. *Physical Review E*, 74(1):016110, 2006.

[8] Jean-Charles Delvenne, Sophia N Yaliraki, and Mauricio Barahona. Stability of graph communities across time scales. *Proceedings of the National Academy of Sciences*, 107(29):12755–12760, 2010.

[9] Alberto Fernández and Sergio Gómez. Versatile linkage: a family of space-conserving strategies for agglomerative hierarchical clustering. *Journal of Classification*, 37(3):584–597, 2020.

Table S1: Partition density of the single-level cut ($D_{\rm lc}$) and the adaptive cut ($D_{\rm ac}$), relative improvement, and balancedness B across four linkage methods and twenty networks drawn uniformly at random (seed 42) from the catalogue with $50 \leq M < 2000$. Networks are sorted by edge count.

Network	Linkage	$D_{\rm lc}$	$D_{\rm ac}$	Improv.	B
high-school-contacts	single	0.446	0.476	+6.8%	0.16
	average	0.268	0.300	+11.9%	0.65
	weighted	0.265	0.275	+3.9%	0.67
	Ward	0.242	0.242	+0.0%	0.67
human-brain-AAL	single	0.435	0.465	+6.8%	0.18
	average	0.331	0.338	+2.0%	0.64
	weighted	0.265	0.274	+3.2%	0.64
	Ward	0.221	0.221	+0.0%	0.65
workplace-contacts	single	0.325	0.333	+2.4%	0.20
	average	0.162	0.162	+0.0%	0.71
	weighted	0.166	0.190	+14.4%	0.73
	Ward	0.232	0.237	+2.0%	0.71
Zachary	single	0.285	0.403	+41.5%	0.33
	average	0.244	0.260	+6.7%	0.48
	weighted	0.214	0.259	+21.0%	0.44
	Ward	0.205	0.278	+35.3%	0.62
star-wars	single	0.321	0.384	+19.6%	0.17
	average	0.230	0.292	+27.3%	0.64
	weighted	0.233	0.294	+26.0%	0.64
	Ward	0.193	0.260	+34.9%	0.65
software-colt	single	0.138	0.173	+25.5%	0.20
	average	0.058	0.083	+42.4%	0.38
	weighted	0.068	0.090	+32.3%	0.43
	Ward	0.063	0.099	+57.2%	0.50
human-brain-CPAC	single	0.249	0.295	+18.4%	0.28
	average	0.167	0.224	+34.3%	0.69
	weighted	0.170	0.214	+26.3%	0.71
	Ward	0.121	0.215	+77.8%	0.70
software-jmail	single	0.224	0.265	+17.9%	0.24
	average	0.136	0.181	+33.2%	0.60
	weighted	0.161	0.196	+22.2%	0.62
	Ward	0.115	0.170	+47.8%	0.69
internet-TataNld	single	0.152	0.173	+14.1%	0.29
	average	0.129	0.147	+14.2%	0.51
	weighted	0.129	0.141	+9.6%	0.58
	Ward	0.129	0.148	+14.3%	0.61
facebook-friends	single	0.414	0.437	+5.7%	0.29
	average	0.354	0.386	+9.1%	0.60
	weighted	0.358	0.401	+12.1%	0.61
	Ward	0.270	0.416	+54.0%	0.65
london-transport	single	0.092	0.111	+20.2%	0.26
	average	0.085	0.088	+4.2%	0.44
	weighted	0.085	0.089	+5.3%	0.44
	Ward	0.091	0.093	+1.3%	0.46
Les Miserables	single	0.577	0.598	+3.8%	0.09
	average	0.525	0.546	+4.0%	0.34
	weighted	0.552	0.571	+3.4%	0.31
	Ward	0.473	0.583	+23.3%	0.37
internet-Kdl	single	0.071	0.076	+6.5%	0.27
	average	0.055	0.065	+17.4%	0.39
	weighted	0.055	0.063	+14.7%	0.32
	Ward	0.055	0.060	+8.9%	0.40
political-books	single	0.287	0.307	+7.1%	0.32
	average	0.233	0.297	+27.3%	0.69
	weighted	0.214	0.260	+21.5%	0.71
	Ward	0.204	0.266	+30.1%	0.75
streets-bologna	single	0.124	0.130	+4.4%	0.25
	average	0.089	0.120	+35.2%	0.41
	weighted	0.087	0.111	+26.2%	0.41
	Ward	0.104	0.110	+5.3%	0.40
unicodelang	single	0.016	0.024	+48.8%	0.08
	average	0.010	0.013	+33.9%	0.38
	weighted	0.009	0.012	+26.5%	0.38
	Ward	0.007	0.011	+52.6%	0.46
software-jung	single	0.163	0.211	+29.8%	0.11
	average	0.143	0.177	+23.7%	0.59
	weighted	0.153	0.179	+17.2%	0.57
	Ward	0.122	0.195	+59.2%	0.65
eu-proc-EE	single	0.006	0.010	+49.5%	0.16
	average	0.005	0.007	+44.9%	0.29
	weighted	0.004	0.005	+24.8%	0.34
	Ward	0.002	0.002	+28.5%	0.32
eu-proc-PT	single	0.004	0.006	+52.6%	0.09
	average	0.003	0.004	+9.1%	0.26
	weighted	0.002	0.002	+17.2%	0.34
	Ward	0.002	0.002	+19.9%	0.35
eu-proc-AT	single	0.002	0.004	+142.0%	0.10
	average	0.002	0.000	−85.0%	0.29
	weighted	0.001	0.001	−9.7%	0.28
	Ward	0.002	0.003	+20.8%	0.26

Table S2: D at 10^3, 10^4, and 10^5 MCMC steps on the five reference networks. All but `euroroad` reach their 10^5-step value to within 0.5% by 10^4 steps (`euroroad` within $\sim 4\%$); for $M < 200$, 10^3 steps is sufficient.

Network	M	D_{lc}	D at 10^3	D at 10^4	D at 10^5
Zachary	78	0.285	0.403	0.403	0.403
contiguous_usa	107	0.277	0.324	0.348	0.348
euroroad	1,305	0.068	0.075	0.083	0.086
foodweb_baywet	2,075	0.299	0.304	0.308	0.308
cintestinalis	2,575	0.165	0.168	0.172	0.174

Table S3: Final D at 5×10^4 steps for each (network, initialisation) pair. k_{start} in parentheses. Bold marks the highest D among the three initialisations for each network.

Network	M	D_{lc}	$k_{\text{start}} = k_{\text{lc}}$	$k_{\text{start}} = M/10$	$k_{\text{start}} = M$
Zachary	78	0.285	**0.403** ($k=22$)	**0.403** ($k=7$)	**0.403** ($k=78$)
Les Miserables	254	0.577	**0.598** ($k=52$)	**0.598** ($k=25$)	**0.598** ($k=254$)
Football	613	0.550	**0.565** ($k=161$)	0.119 ($k=61$)	**0.565** ($k=613$)
euroroad	1,305	0.068	**0.086** ($k=1053$)	**0.086** ($k=130$)	**0.086** ($k=1305$)
foodweb_baywet	2,075	0.299	**0.308** ($k=81$)	**0.308** ($k=207$)	0.289 ($k=2075$)
cintestinalis	2,575	0.165	**0.172** ($k=663$)	0.143 ($k=257$)	0.143 ($k=2575$)
Jazz	2,742	0.416	0.435 ($k=223$)	0.435 ($k=274$)	**0.437** ($k=2742$)
bible_nouns	9,059	0.178	**0.292** ($k=3372$)	0.140 ($k=905$)	0.285 ($k=9059$)

Table S4: Height-based stress test on `foodweb_baywet`: chain initialised at a height-0.4 cut ($k_{\text{start}} = 562$, $D = 0.149$ at $t = 0$), 10^6 steps, $T = 10^{-4}$, three independent seeds. All trials climb back to within 0.06 of $D_{\text{lc}} = 0.299$, with one trial within 0.002.

Trial	k_{start}	D at $t = 0$	D at $t = 10^6$
0	562	0.149	0.244
1	562	0.149	0.260
2	562	0.149	0.297

Table S5: Heuristic T_0 from Eq. S5.1 for the eight networks of Fig. S4.

Network	M	B	D_{lc}	T_0 (heuristic)
Zachary	78	0.325	0.285	1.0×10^{-3}
Les Miserables	254	0.092	0.577	1.0×10^{-3}
Football	613	0.194	0.550	1.6×10^{-4}
euroroad	1,305	0.238	0.068	7.7×10^{-5}
foodweb_baywet	2,075	0.318	0.299	4.8×10^{-5}
cintestinalis	2,575	0.279	0.165	3.9×10^{-5}
Jazz	2,742	0.195	0.416	3.7×10^{-5}
bible_nouns	9,059	0.148	0.178	3.3×10^{-5}

Table S6: Single-process wall-clock benchmark of adaptive cut. Networks ordered by M. *Dendro.* is the time to read the edge list, compute Jaccard similarities, and run single-linkage; *MCMC* is 2×10^5 steps of the adaptive-cut sampler at $T = 10^{-4}$, seed 42.

Network	M	D_{lc}	D_{mcmc}	Dendro. (s)	MCMC (s)
Zachary	78	0.285	0.403	0.01	3.20
contiguous_usa	107	0.277	0.348	0.01	3.52
Les Miserables	254	0.577	0.598	0.02	3.75
Football	613	0.550	0.565	0.05	8.80
euroroad	1,305	0.068	0.086	0.06	16.12
foodweb_baywet	2,075	0.299	0.308	1.07	7.91
cintestinalis	2,575	0.165	0.174	1.12	24.02
Jazz	2,742	0.416	0.435	1.21	12.84
bible_nouns	9,059	0.178	0.294	6.20	183.67
us_congress-H93	18,083	0.179	0.179	63.68	18.00

Table S7: Improvement of adaptive cut over single-level cut, binned by balancedness B.

B bin	n	mean $\Delta D/D_{\mathrm{lc}}$ (%)	median (%)	$P(\Delta D \geq 5\%)$	$P(\Delta D \geq 10\%)$	$P(\Delta D \geq 25\%)$
[0.00, 0.10)	54	52.6	56.8	0.87	0.78	0.61
[0.10, 0.15)	45	33.0	23.2	0.78	0.78	0.47
[0.15, 0.20)	40	30.3	25.0	0.80	0.65	0.50
[0.20, 0.25)	27	24.3	17.9	0.85	0.78	0.44
[0.25, 0.30)	24	16.4	13.2	0.83	0.54	0.12
[0.30, 0.50)	12	19.0	14.5	0.83	0.67	0.33

Table S8: Pearson and Spearman correlations of structural metrics with balancedness B, with 95% bootstrap confidence intervals over 1000 resamples ($n = 195$ networks where both partitions and the dendrogram are available).

Metric	Pearson r (95% CI)	Spearman ρ (95% CI)
$1 - \mathrm{NMI}(\sigma_{\mathrm{lc}}, \sigma_{\mathrm{ac}})$	−0.53 (−0.62, −0.43)	−0.42 (−0.55, −0.28)
$\mathrm{Colless}_{\mathrm{norm}}$	+0.31 (+0.18, +0.44)	+0.31 (+0.18, +0.42)
$\mathrm{Sackin}_{\mathrm{norm}}$	+0.37 (+0.24, +0.50)	+0.37 (+0.24, +0.48)
$\log_{10}(\max m_c / \min m_c)$ in σ_{lc}	−0.28 (−0.41, −0.13)	−0.27 (−0.41, −0.12)

Table S9: Mean B, k_{lc}, and edge count M over 5 seeds in each cell of the 2×2 design.

cell	density	structure	B (mean ± std)	k_{lc} (mean ± std)	M (mean)
(A)	uniform	flat	0.237 ± 0.055	38.8 ± 20.6	283
(B)	varying	flat	0.144 ± 0.036	54.8 ± 14.0	285
(C)	uniform	nested	0.273 ± 0.042	36.6 ± 60.1	356
(D)	varying	nested	0.151 ± 0.027	97.0 ± 26.2	358

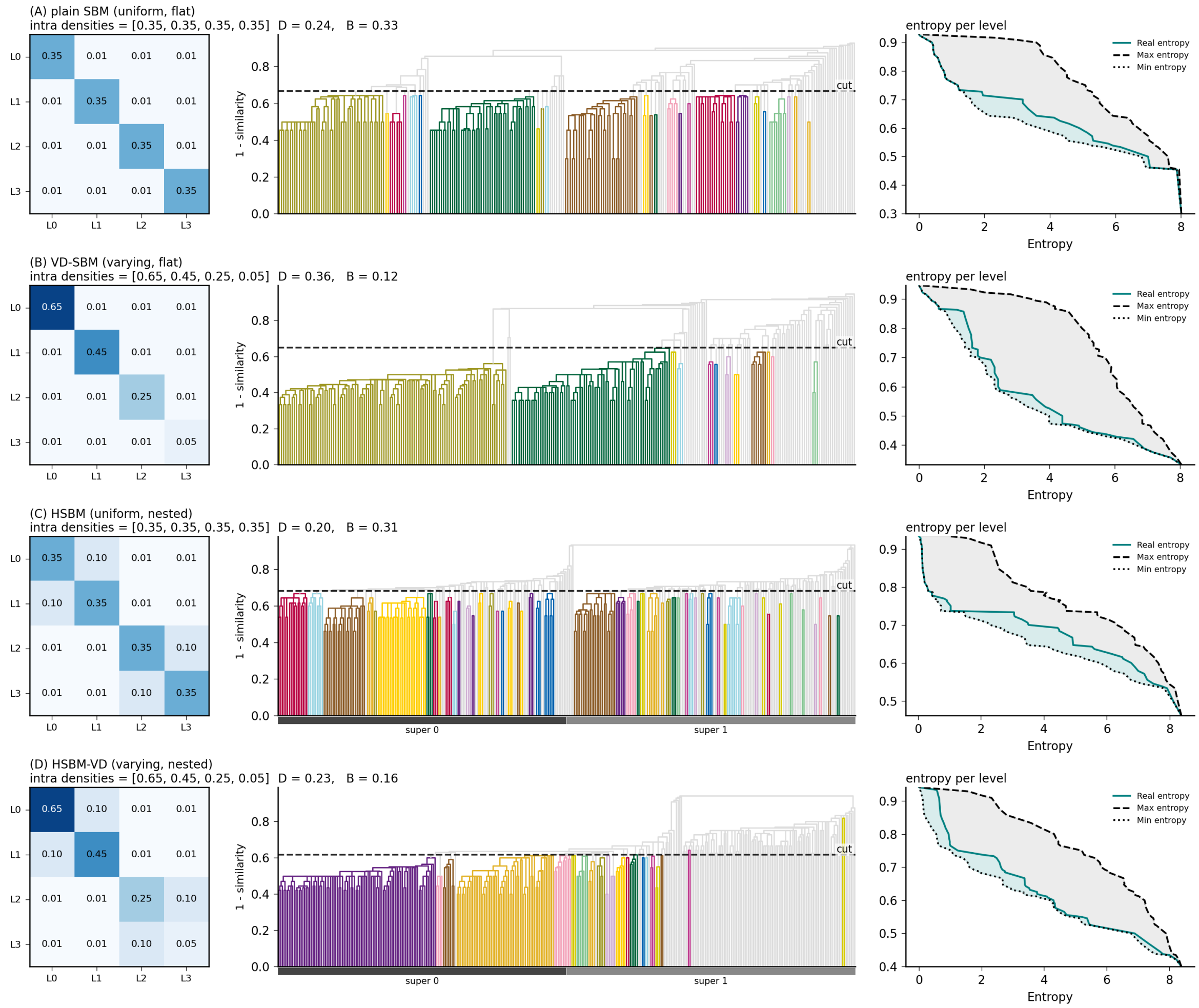

Figure S7: 2×2 design crossing uniform vs varying intra-community density (columns) against flat vs 2-level nested community structure (rows), seed 0. For each cell: block-density matrix, dendrogram coloured by σ_{lc} with the optimal cut as a dashed line, and entropy curves $H_{\min} \leq H_{\mathrm{real}} \leq H_{\max}$ vs similarity. The drop in B along the density-variation axis (A→B and C→D) is visible as the real entropy curve pulling down to $H_{\min}$. The nesting axis (A→C and B→D) does not move the entropy curve appreciably.

Table S10: Mean B across 5 seeds in each cell of the 2×2 design (8 leaves, 3-level nesting), under three linkage methods applied to the same Jaccard edge-distance matrix per draw.

cell	density	structure	B_{single}	B_{average}	B_{complete}
(A)	uniform	flat	0.132 ± 0.033	0.663 ± 0.017	0.591 ± 0.022
(B)	varying	flat	0.155 ± 0.042	0.606 ± 0.014	0.568 ± 0.013
(C)	uniform	3-level nested	0.131 ± 0.025	0.693 ± 0.012	0.624 ± 0.015
(D)	varying	3-level nested	0.120 ± 0.045	0.651 ± 0.023	0.600 ± 0.024

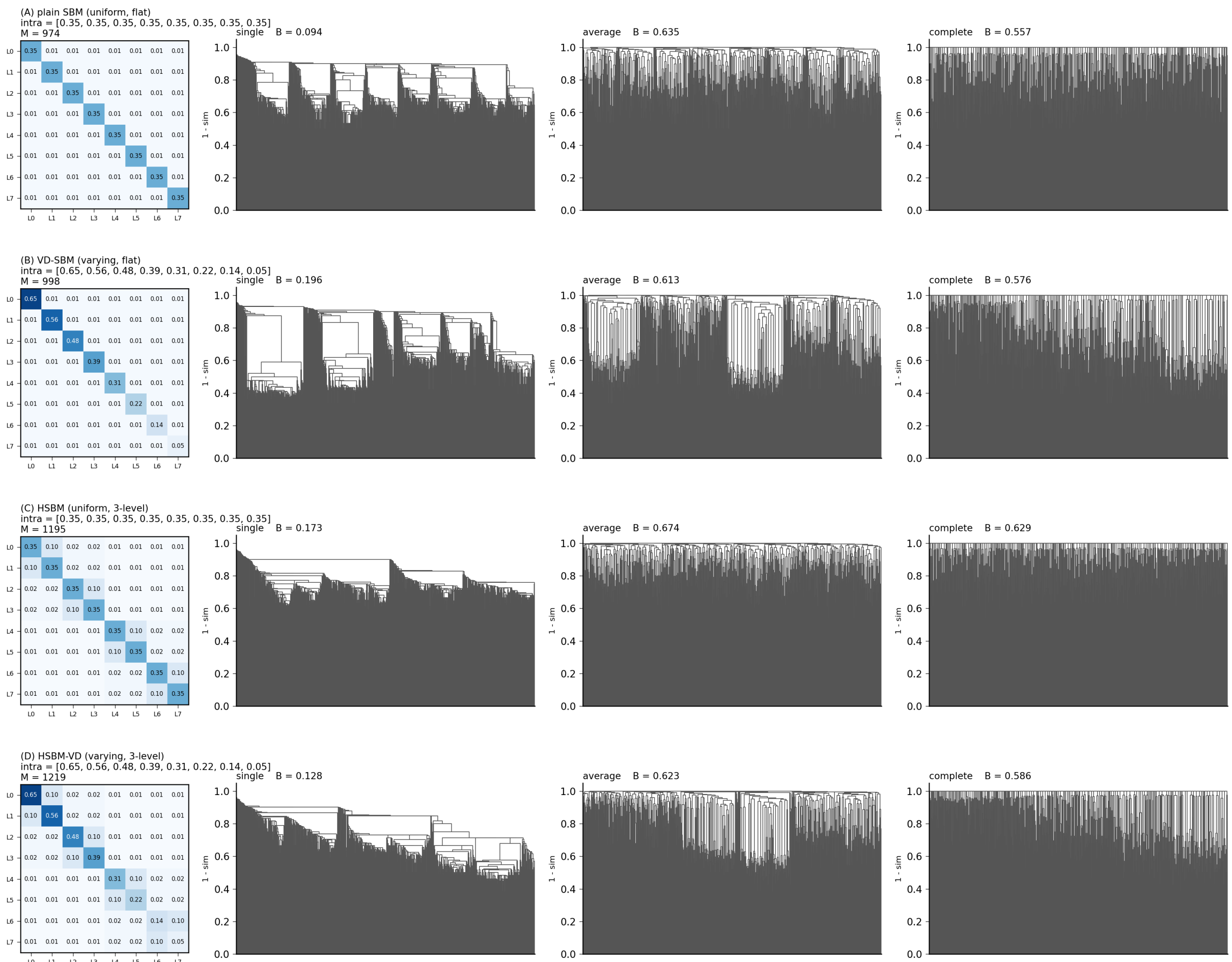

Figure S8: Linkage sensitivity of B on the 8-leaf, 3-level 2×2 design. Each row is a cell (A, B, C, D); the first column shows the SBM block-density matrix; the remaining three columns show the dendrogram and B value when single, average, or complete linkage is applied to the same Jaccard edge-distance matrix. Single linkage on this larger graph chains and gives $B \in [0.12, 0.16]$ across all cells, masking the cell-to-cell signal. Under both average and complete linkage the uniform-density cells (A, C) score above their density-varying counterparts (B, D) at a higher absolute scale.

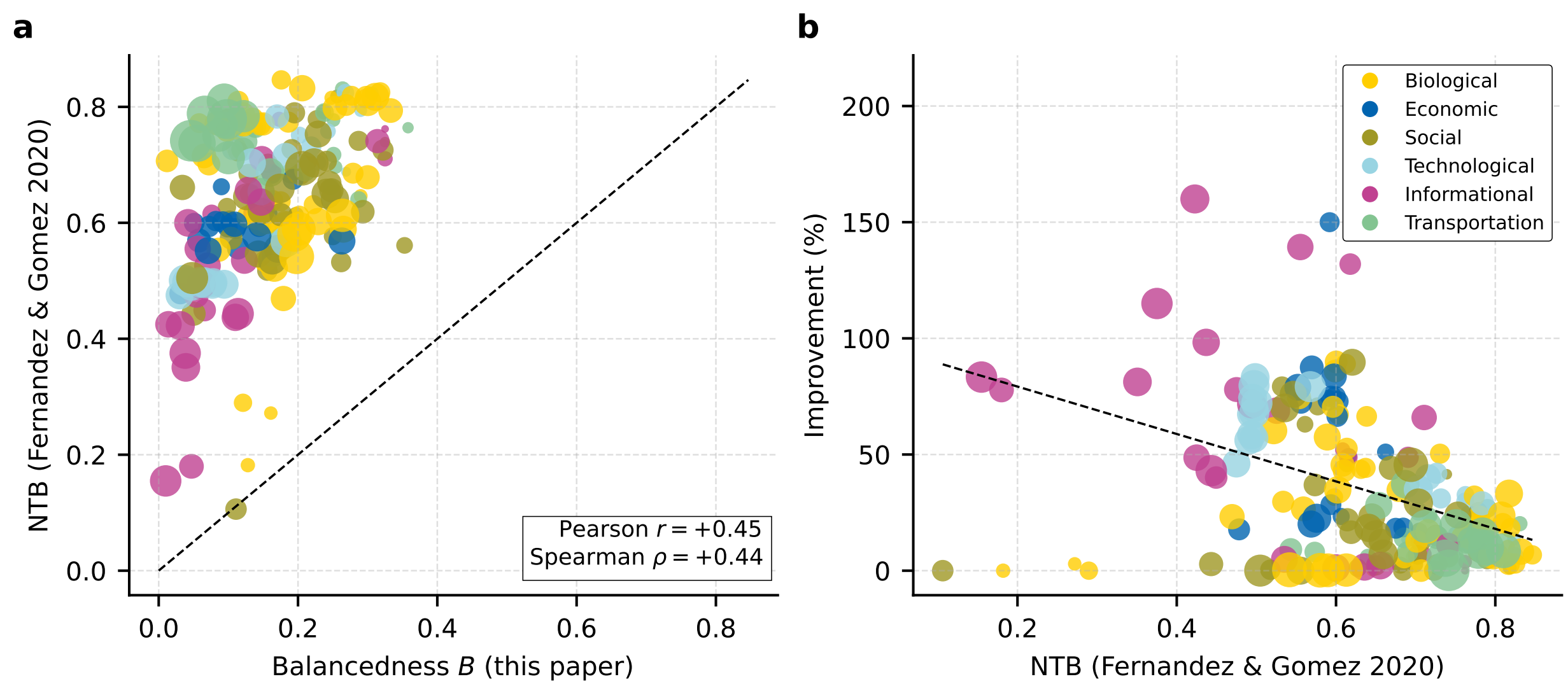

Figure S9: Comparison of our balancedness B with the Fernández–Gómez 2020 normalised tree balance (NTB). (a) B vs NTB on 202 catalogue networks (Pearson $r = +0.45$, Spearman $\rho = +0.44$); the two indices are correlated but distinct. (b) Adaptive-cut improvement over D_{lc} vs NTB, reproducing the Fig. 4 scatter with NTB on the x-axis. Pearson correlations of improvement with the two balance indices are nearly identical: $r(\mathrm{impr}, B) = -0.38$ and $r(\mathrm{impr}, \mathrm{NTB}) = -0.40$ ($n = 202$).

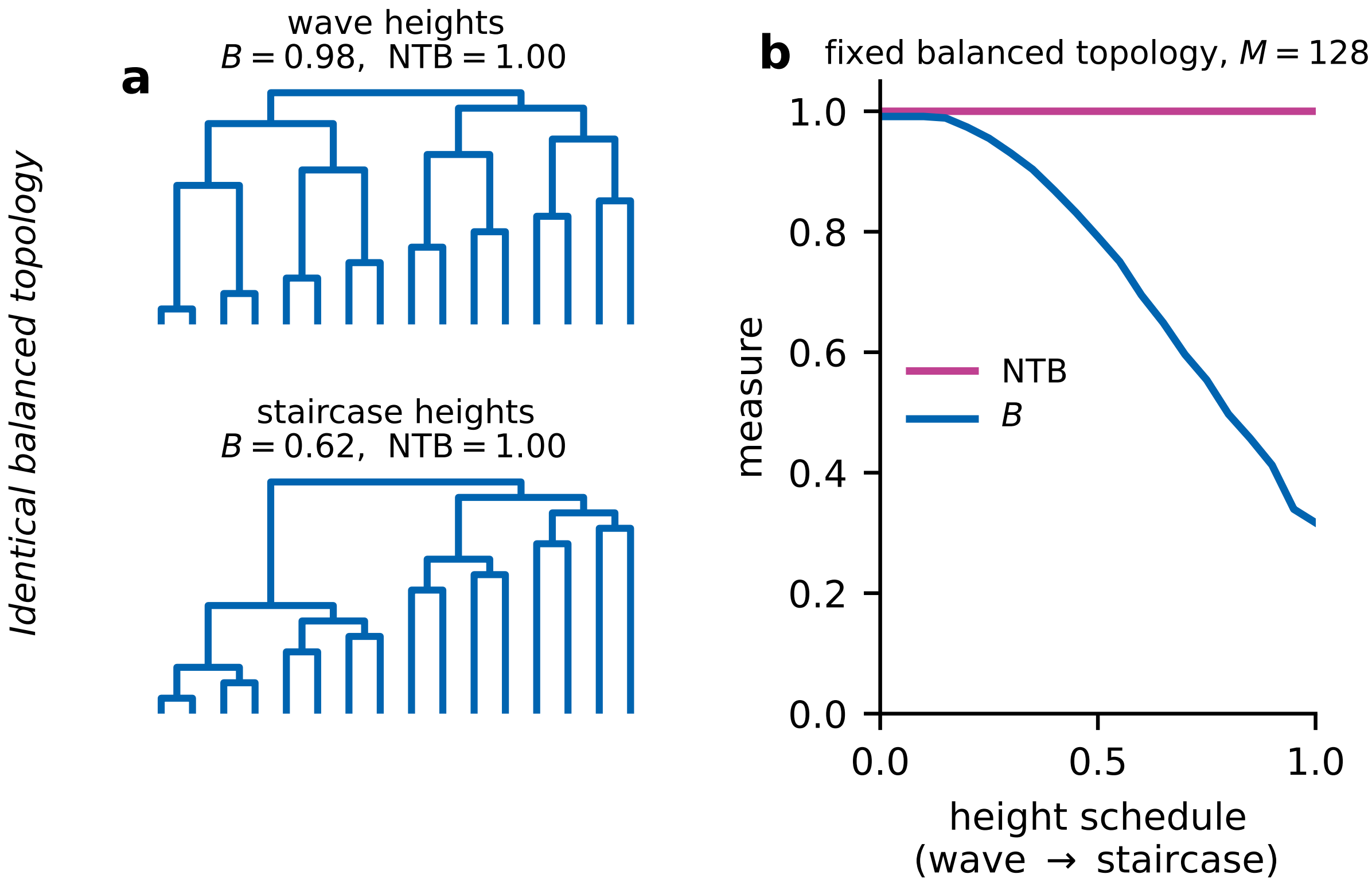

Figure S10: Balancedness B and NTB capture contrasting dendrogram properties. (a) Two dendrograms with the *same* balanced binary topology (NTB = 1 for both, $M = 16$): merging in synchronised waves keeps every horizontal cut balanced ($B = 0.98$), while a staircase height schedule makes each cut dominated by one growing cluster ($B = 0.62$). (b) Holding the balanced topology fixed at $M = 128$ and interpolating the height schedule from waves to staircase moves B from 0.99 to 0.32 while NTB stays at 1: NTB is blind to merge heights, B is not.
